# Cation Vacancies with and without O–O Dimerization: Identifying Reconstructed Defect Configurations in Metal Oxides

**DOI:** 10.1021/jacs.6c15378

**Published:** 2026-10-07

**Authors:** Taifeng Liu, Xingfan Zhang, Jamal Abdul Nasir, Qing Hou, Liam Morgan, Jingcheng Guan, Thomas Durrant, You Lu, Thomas W. Keal, John Buckeridge, Scott M. Woodley, Stefan Goedecker, C. Richard A. Catlow, Alexey A. Sokol

**Affiliations:** 1 National & Local Joint Engineering Research Center for Applied Technology of Hybrid Nanomaterials, Henan University, Kaifeng 475004, P. R. China; 2 Kathleen Lonsdale Materials Chemistry, Department of Chemistry, 4919 University College London, 20 Gordon Street, London WC1H0AJ, United Kingdom; 3 Department of Physics, 27209 University of Basel, Klingelbergstrasse 82, CH-4056 Basel, Switzerland; 4 Institute of Photonic Chips, University of Shanghai for Science and Technology, 580 Jungong Road, Shanghai 200093, China; 5 Scientific Computing Department, STFC Daresbury Laboratory, Warrington WA4 4AD, Cheshire, United Kingdom; 6 School of Engineering and Design, 4914 London South Bank University, 103 Borough Road, London SE1 OAA, United Kingdom; 7 School of Chemistry, Cardiff University, Park Place, Cardiff CF10 1AT, United Kingdom

## Abstract

Point defects in solid-state materials are conventionally modeled as lattice-derived defects, generated by adding or removing atoms from symmetric bulk lattice structures, followed by local geometry optimization. This approach often yields symmetric, weakly relaxed local minima that remain close to the parent lattice. Here, we show that this assumption can fail qualitatively for vacancies in many metal oxides. Rather than retaining a lattice-derived geometry with localized holes on oxygen sites, cation vacancies can drive extensive reconstruction, producing defect configurations in which the O–O bonds and O–O–O chains become the preferred charge-compensation motifs. We combined the minima hopping (MH) structure prediction algorithm with machine-learned interatomic potentials (MLIPs), hybrid QM/MM embedded-cluster calculations, and periodic density functional theory (DFT) calculations to investigate cation vacancies across a broad series of metal oxides. Our results show a clear oxidation-state-dependent trend. In the charge-neutral state, cation vacancies in oxides with formal oxidation states of +4 or higher favor reconstructed defect configurations containing O–O bonds or O–O–O chains with bond lengths of approximately 1.4 Å, lowering the defect formation energy by up to several electronvolts relative to the conventional structure. By contrast, in oxides with lower cation oxidation states, such reconstructions are generally unfavorable at charge neutrality but can become energetically more favorable at positive charge states. These findings define oxygen-bond formation as a general reconstruction pathway for cation vacancies in metal oxides and show that global structure searches are essential for predictive defect modeling beyond conventional lattice-derived local minima.

## Graphical abstract

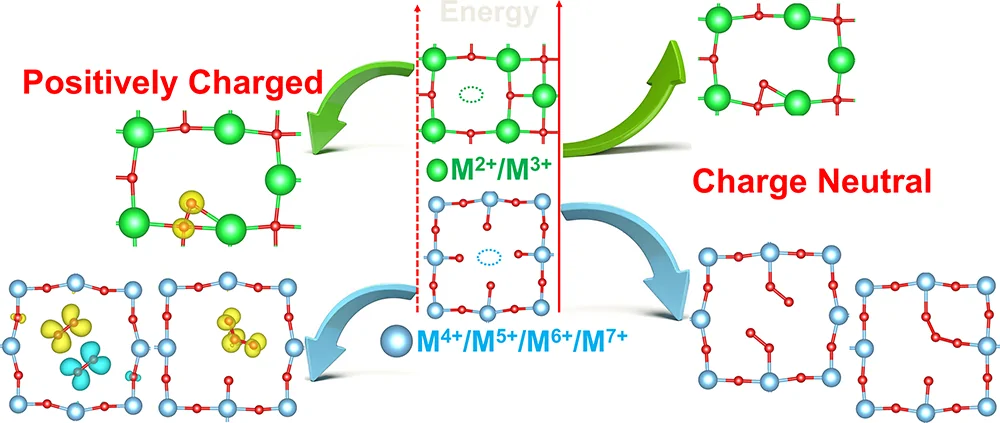

## Introduction

In functional materials, many physical properties are governed by defects rather than by ideal bulk structures.^1^ Point defects in solid-state materials can give rise to electronic and ionic conductivity,^2^ generate catalytic active centers,^3^ or enable information storage through localized spin states.^4,5^ These defects play a crucial role in various technological applications, including photo­(electro)­chemistry, electronic devices, and heterogeneous catalysis.^6–8^ Understanding the properties of defects is therefore essential to the rational design of functional materials.^9^

In metal oxides, point defects such as vacancies are often assumed to adopt a symmetric configuration, in which removal of an atom from the lattice site is followed only by modest local relaxation. However, such structures may correspond only to a local minimum on the potential energy surface and therefore do not necessarily account for experimentally measured transport, optical, magnetic, or catalytic properties. Therefore, identifying reconstructed defect configurations far from ideal lattice structures and determining the true ground state, or an ensemble of thermodynamically accessible states, of a point defect is critical.^10^ An early demonstration of this principle was provided by Goedecker et al.,^11^ who systematically explored the configurational space of defects in silicon, and identified a 4-fold-coordinated defect with a lower formation energy than conventional defect types, including vacancies, interstitials, and Frenkel pairs. This defect structure represents a substantial departure from standard point-defect models. Another representative example is the copper vacancy in Cu_2_O. Scanlon et al.^12^ reported that two types of copper vacancies exist: a conventional *V*_Cu_, formed by removing one Cu atom and leaving two three-coordinated oxygen atoms, and *V*_Cu_^Split^, where a remaining Cu atom moves toward the vacancy site to achieve a tetrahedral coordination. The *V*_Cu_^Split^ was identified as the lower-energy configuration, although the conventional *V*_Cu_ is also expected to be present in experimental samples due to the low energy difference. Mosquera-Lois et al.^13,14^ predicted that in crystals with stronger ionic character, defect reconstructions can be driven either by homoionic bond formation (e.g., peroxide formation in In_2_O_3_, ZnO and TiO_2_) or by Jahn–Teller/crystal field stabilization effects (e.g., in CeO_2_). The same group developed the ShakeNBreak code,^10,13,15^ a defect structure-searching method that employs chemically guided bond distortions to locate ground-state and metastable structures of point defects in solid materials. In SrO, our previous work^16^ demonstrated that in the charge-neutral singlet state, a peroxide-like oxygen interstitial, in which the interstitial oxygen forms an O–O bond with a lattice oxygen atom, has a formation energy approximately 2 eV lower than that of the conventional configuration. These studies show that dimer reconstructions involving oxygen interstitials are a common reconstruction pathway in metal oxides.^17,18^

Dimer and trimer reconstructions involving anion–anion bond formation are also reported for metal vacancies in metal sulfides and selenides, such as Sb_2_S_3_ and Sb_2_Se_3_. The formation of S–S or Se–Se dimers serves as a charge compensation mechanism, facilitated by the low electronegativity and high polarizability of S and Se.^13^ In contrast, for cation vacancies in oxide materials, analogous reconstructions that lead to O–O bond formation remain poorly understood. Sokol et al.^19,20^ first reported O–O bond formation associated with silicon vacancies in microporous aluminosilicates, a finding that was later also confirmed in quartz.^21^ However, this phenomenon has not been systematically explored in other oxides. Global structure prediction of charged defects at the density functional theory (DFT) level remains computationally expensive. Traditional interatomic potentials can reduce the cost, but they often lack sufficient accuracy for charged defects unless they are carefully parametrized for the relevant charge states, local coordination environments, and long-range electrostatic interactions.^22^ Currently, widely used periodic electronic-structure codes and machine learning interatomic potentials (MLIPs) are more reliable for charge-neutral defects than for highly charged defects,^15^ where artificial image–image interactions and atomic relaxation under finite supercells remain insufficiently described. Nevertheless, MLIPs^23^ are highly effective for the broad exploration of neutral defect configurations. When combined with structure prediction algorithms, they provide an efficient route to sample the large configurational space and identify low-energy defect structures for subsequent validation using higher-level electronic-structure methods.

In this study, we systematically investigated both neutral and charged configurations of cation vacancies across a range of metal oxides using a combination of several computational methods. First, we employed the minima hopping (MH) structure prediction algorithm^24,25^ combined with machine-learned interatomic potentials^26–28^ to search for low-energy configurations of charge-neutral cation vacancies. This global-search step enables the identification of distinct local minima among candidate structures, including configurations with isolated O–O bonds, paired O–O bonds, and O–O–O chains. Second, guided by the MH results, we examined representative oxides with cation oxidation states of +4, +3, and +2 using the hybrid quantum mechanical/molecular mechanical (QM/MM)^29–32^ embedded-cluster approach, which allows both neutral and charged defect states to be treated with high-level electronic-structure theory. Finally, based on both the MH and QM/MM results, we investigated the charge-neutral reconstructed configurations of metal vacancies across a broader set of common metal oxides using periodic DFT. This integrated strategy shows that O–O and O–O–O formation at cation vacancies is a systematic reconstruction pathway controlled by cation oxidation state and defect charge state, thereby redefining how point defects in metal oxides should be modeled beyond normal lattice-derived geometries.

## Results and Discussion

### Global Search of Hidden Reconstructions Associated with Cation Vacancy Formation with Minima Hopping

We first employ the minima hopping (MH) algorithm combined with the MACE-OMAT-0 ^26^ foundation MLIP to search for possible distinct configurations of charge-neutral metal vacancies in metal oxides. The materials considered span formal cation oxidation states from +1 to +6 and coordination numbers of 4, 6, 7, and 8. These include Cu_2_O, ZnO, Al_2_O_3_, CeO_2_, V_2_O_5_, and WO_3_ (a–f), among others (MgAl_2_O_4_, MnO, Mn_2_O_3_, Mn_3_O_4_, MnO_2_, SrO, SrTiO_3_, TiO_2_, and MgO) in the Supporting Information, all of which are widely used in various technological fields. The MH simulations identified tens to hundreds of distinct local minima, indicating a broad configurational landscape extending beyond the conventionally assumed lattice-derived vacancy structures, particularly in oxides with formal cation oxidation states of +4 or higher. The MH results for some representative oxides are discussed below, with additional systems provided in Figures S1–S12 in the Supporting Information, and the computational details are provided in the methodology below.

Figure 1a–f shows the density of configurations (DOC) as a function of the relative energy for the neutral cation vacancies sampled by MH. In each material, MH identifies multiple structurally distinct local minima. In addition to conventional vacancy configurations without O–O bonds (which may exhibit slight distortions), several reconstructed configurations containing O–O bonds are identified.

**Figure 1:**
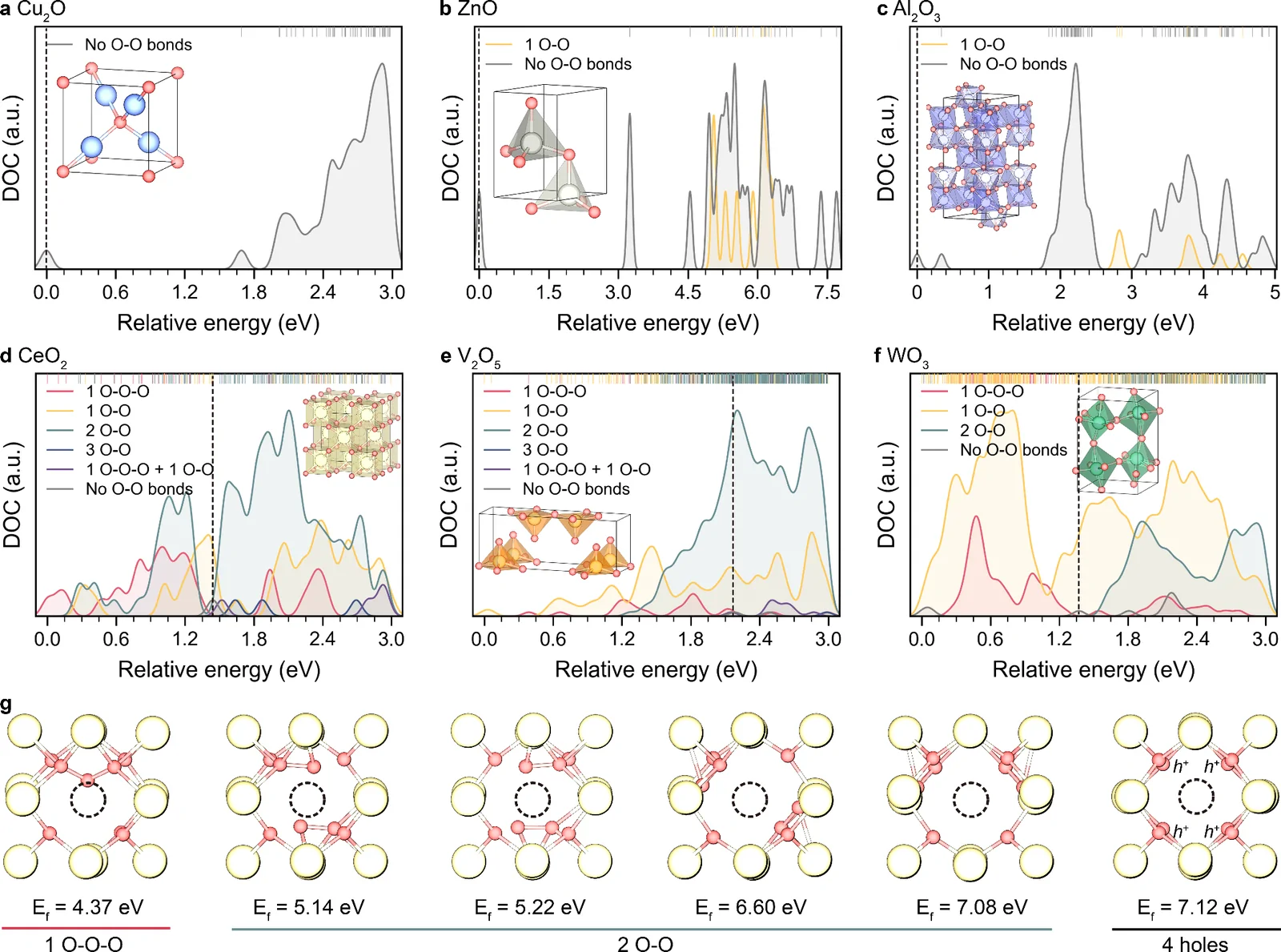
Density of configurations (DOC) as a function of relative energy sampled by the MH simulations for cation vacancies in (a) Cu_2_O, (b) ZnO, (c) Al_2_O_3_, (d) CeO_2_, (e) V_2_O_5_, and (f) WO_3_. The relative energies are referenced to the lowest-energy defect configuration identified for each material. Vertical dashed lines indicate the conventional configurations obtained from local relaxation of the lattice-derived structures. (g) Representative Ce-vacancy configurations in CeO_2_ identified by MH searches and subsequently confirmed by hybrid QM/MM calculations. Both the most stable O–O–O chain configuration and several reconstructed configurations containing two O–O bonds have lower formation energies than the conventional quasi-symmetric vacancy configuration with four localized holes.

For oxides with lower cation oxidation states, such as Cu_2_O (+1), ZnO (+2), MgAl_2_O_4_ (both V_Al_ (+3) and V_Mg_ (+2)), SrTiO_3_ (V_Sr_ (+2)), and Al_2_O_3_ (+3), the reconstructed structures could contain only a single O–O bond. For Cu_2_O, such reconstructed O–O-bonded configurations are much higher in energy (>6 eV) and are therefore not shown in the figure. Similarly, in ZnO and Al_2_O_3_, while the O–O-containing configurations are found by MH, they lie in relatively high-energy regions and are therefore thermodynamically not accessible.

In contrast, oxides with higher formal oxidation states, including CeO_2_ (+4), SrTiO_3_ (V_Ti_ (+4)), V_2_O_5_ (+5), and WO_3_ (+6), show an entirely different picture. In general, two major types of reconstructions are identified: those with peroxide-like O–O bonds and those with an O–O–O chain. These motifs span a broad region of the energy landscape because O–O bond formation can occur at different positions and orientations relative to the cation-vacancy site. Moreover, hole localization, O–O bond formation, and O–O–O chain formation can operate together to compensate the charge imbalance created by cation vacancies. For example, in CeO_2_, a configuration containing one O–O bond together with two localized holes can be obtained, although this configuration with one O–O bond lies slightly higher in energy than the most stable O–O–O chain configuration. As a result, higher formal cation oxidation states promote a more complex configurational landscape associated with cation vacancies, as captured by the MH searches. Across these reconstructed structures, the O–O bond lengths are approximately 1.4 Å. Many of these configurations lie in relatively low-energy regions and are substantially more stable than the conventional symmetric vacancy configuration, indicating that oxygen dimerization is an energetically favorable reconstruction pathway associated with cation vacancy formation in many metal oxides.

These results motivate a systematic examination of oxygen reconstruction around undercoordinated oxygen atoms at cation vacancies across oxides with different metal oxidation states. As MACE-OMAT-0 is a foundation MLIP, the predicted relative energies of different defect configurations may contain material-dependent errors. Hence, the MH results were used primarily as a structure-discovery tool to generate candidate reconstructions that would be difficult to anticipate from chemical intuition alone. As shown below, however, the MH searches at this level already capture the general oxidation-state-dependent trend that is later confirmed by our hybrid QM/MM and plane-wave DFT calculations. Guided by these results, we next examine the representative O–O and O–O–O vacancy configurations using the QM/MM model, as well as extending beyond the charge-neutral case.

### QM/MM Analysis of Charge-State Preference of O–O- and O–O–O-type Reconstructions of Cation Vacancies

From a chemical perspective, the removal of a metal ion not only creates undercoordinated oxygen atoms but also introduces holes into the system. As noted above, these holes can localize on oxygen sites and promote the reconstruction of oxygen atoms, leading to the formation of O–O bonds or O–O–O chains.

In this section, we employ the state-of-the-art hybrid QM/MM embedded-cluster approach to model accurately the reconstruction of oxygen atoms associated with cation vacancy formation. We first use CeO_2_ as a test case to validate these MH-derived configurations at the neutral charge state under high-level QM/MM theory. The hybrid meta-GGA functional BB1K was used for the QM region to improve the description of hole localization in metal oxides and to reduce self-interaction error. A well-parametrized shell-model potential for CeO_2_ was employed in the MM region. This QM/MM setup has been systematically benchmarked for CeO_2_ in our previous studies.^22^ The geometries of the calculated configurations are shown in Figure 1g. Consistent with the MH results, the O–O–O chain configuration is the lowest-energy structure, lying 2.75 eV below the conventional configuration with four holes. Configurations containing two O–O bonds are also accessible, but their stability depends strongly on the relative orientation of the two peroxide-like units around the *V*_Ce_ site. The most favorable two-O–O-bond configuration is 1.98 eV lower in formation energy than the conventional symmetric vacancy, whereas the least favorable arrangement, with two nearest-site O–O bonds, is only 0.04 eV lower. This strong orientational dependence indicates that O–O bond formation alone is insufficient to define the stability of the reconstructed vacancy; the spatial arrangement of the oxygen dimers is also important.

We then extended the QM/MM calculations to a set of representative oxides covering common cation oxidation states and coordination environments. These materials effectively span the common oxidation states of metals in oxides: +4, +3, and +2. Specifically, tetragonal SrTiO_3_ and cubic CeO_2_ are chosen to represent metals in the +4 oxidation state, MgAl_2_O_4_ for the +3 state, and ZnO, SrO, and MgO for the +2 state. These materials also include cation coordination numbers of four, six, and eight, thereby sampling common local environments in metal oxides. Both neutral and charged states (ranging from −2 to +4) are investigated. Details of the QM/MM calculations are shown in the methodology below, and parameters are provided in the Supporting Information.

We next varied the defect charge state to test whether the reconstructed oxygen motifs can accommodate additional or fewer holes, and how this charge accommodation affects their relative stability using SrTiO_3_ as an example. Figure 2a shows the spin density of a “quasi-symmetric” Ti vacancy in SrTiO_3_, where the four excess holes are localized on the four undercoordinated oxygen atoms around the vacancy. In this case, the nearest O–O distance around the vacancy is approximately 2.95 Å. After reconstruction, two types of structures are formed: one with two separated O–O bonds (Figure 2b) and one with an O–O–O chain (Figure 2c). In both cases, the two excess holes are consumed by the O–O bonds, leaving no residual spin density. The O–O bond lengths in these species are approximately 1.40 Å, consistent with the MH results. Upon adding one additional hole (i.e., charge state +1), the two-separated-O–O configuration accommodates the extra hole on one of the two O–O bonds while the other remains unchanged (Figure 2d). In the O–O–O chain configuration, however, the excess hole is distributed over the entire chain (Figure 2e). In both cases, the localization of an extra hole shortens the O–O bonds to approximately 1.30 Å, and the Ti–O bonds elongate from about 2.0 Å to 2.4 Å. Upon adding a further hole to reach charge state +2, the two-O–O configuration can accommodate the second additional hole on the remaining O–O bond, which also shortens to approximately 1.30 Å (Figure 2f). By contrast, the O–O–O chain configuration can no longer accommodate additional holes and remains essentially unchanged. At even higher charge states (+3 and +4), the excess holes do not further affect these species; instead, they localize on other lattice O^2–^ sites as those in the conventional case. These results show that each isolated O–O bond (O_2_^2–^), or each O–O–O chain (O_3_^4–^), can accommodate at most one additional hole (becoming O_2_^3–^ and O_3_^5–^, respectively).

**Figure 2:**
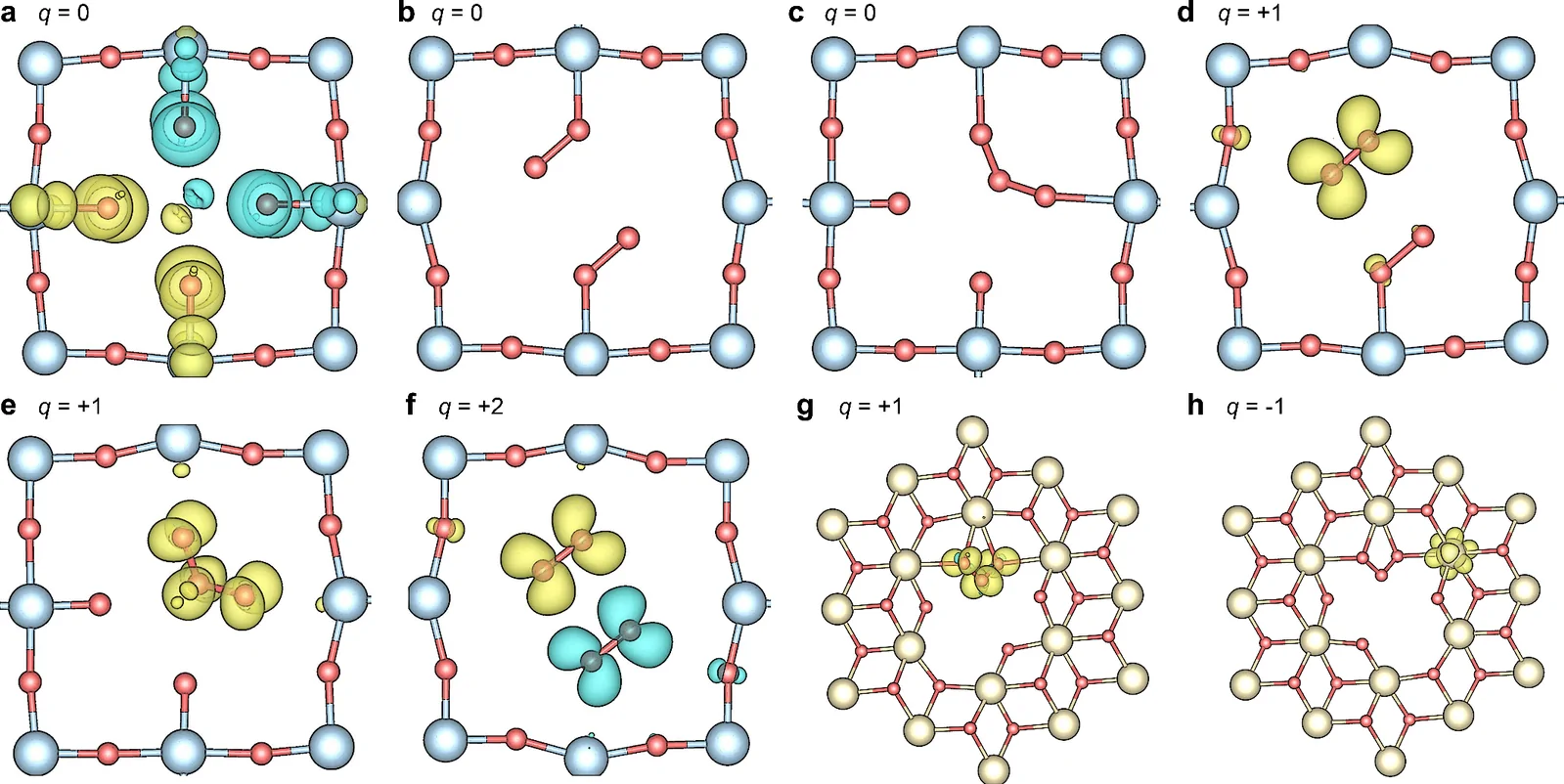
Spin density distributions associated with cation vacancies at different charge states. SrTiO_3_: (a) conventional weakly relaxed configuration (neutral); (b) reconstructed configuration with two O–O bonds (neutral); (c) reconstructed configuration with an O–O–O chain (neutral); (d) two O–O bonds (+1 state); (e) O–O–O chain (+1 state); (f) two O–O bonds (+2 state). CeO_2_: (g) O–O–O chain (+1 state); (h) O–O–O chain (−1 state). Positive and negative spin-density isosurfaces are shown in yellow and cyan, respectively, at values of 0.003 *e* bohr^–3^. The small red spheres represent oxygen atoms, while the other spheres denote metal atoms.

A similar charge-compensation mechanism is observed in CeO_2_. For the most stable charge-neutral O–O–O chain configuration, no spin density is observed as shown in Figure 1g. This closed-shell O_3_^4–^ chain can accommodate an additional hole (Figure 2g), similar to SrTiO_3_. In negatively charged states, however, a different behavior emerges: the excess electron localizes on the Ce site, forming an f-type small electronic polaron (Figure 2h) while leaving the geometry and bonding of the O–O species unaffected. This pathway is energetically preferred because Ce^3+^ polaron formation in CeO_2_ is strongly stabilized by lattice relaxation, with a relaxation energy of approximately 1.4 eV.^33^ Electron addition is therefore accommodated by metal-centered reduction rather than by removal of a hole from the stable O_3_^4–^ chain-like species.

The lower-oxidation-state oxides show a more restricted reconstruction chemistry for cation vacancies. For MgAl_2_O_4_, only the Al vacancy was considered, as Al is formally in the +3 oxidation state. We examined three types of configurations at the QM/MM level of theory: one with an isolated O–O bond (Figure 3a), one with an O–O–O chain (Figure 3b), and one with two O–O bonds (Figure 3c). At the neutral charge state, only the isolated O–O bond configuration can form, while the other two are unstable, consistent with the MH results obtained using the MACE MLIP. For oxides with divalent cations, ZnO, SrO, and MgO, we only focused on the isolated O–O bond configuration. Configurations with two O–O bonds turned out to be higher in energy than the conventional case even at the +2 charge state. For SrO and MgO, which adopt similar rock-salt structures, only one configuration was considered; the structure for SrO is shown in Figure 3d as a representative example. In ZnO, which adopts a tetrahedral cation coordination, the zinc vacancy leaves four undercoordinated oxygen atoms. We therefore considered two distinct orientations, as shown in Figure 3e,f. The corresponding spin density distributions for SrO, MgO, and ZnO are provided in the Supporting Information (Figures S13, S14, and S15–S16, respectively). For MgAl_2_O_4_, only the isolated O–O bond configuration is stable at the neutral charge state. At charge state +1, both the O–O–O chain and the two-O–O configurations become stable. However, in this case, the system contains an even number of electrons, meaning that the excess charge cannot be observed in the spin density. Upon further increasing the charge state, the excess charge localizes on other atomic sites, as each O–O species can accommodate at most one excess hole.

**Figure 3:**
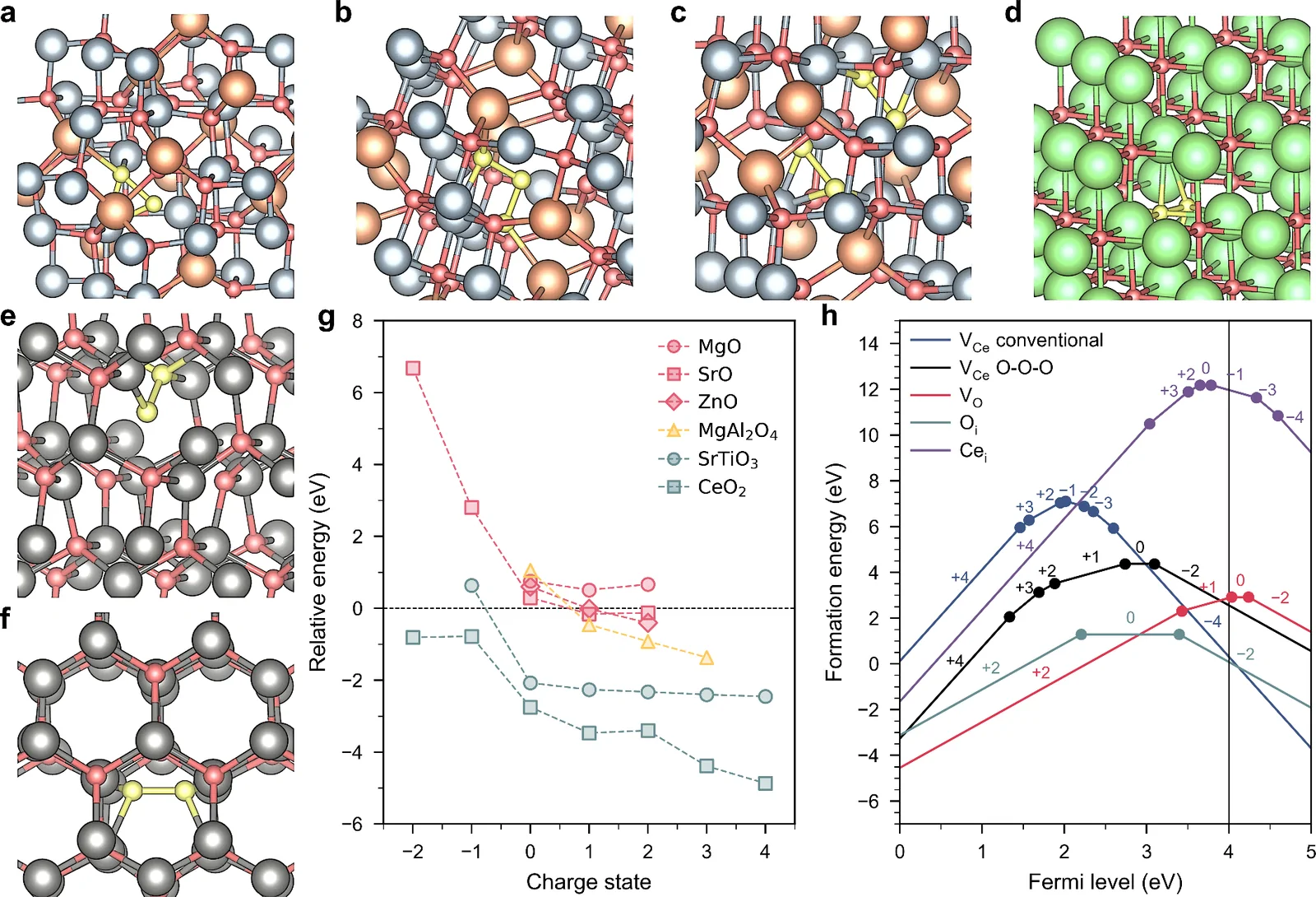
(a–c) Configurations of the Al vacancy in MgAl_2_O_4_: (a) with one O–O bond, (b) with an O–O–O chain, and (c) with two O–O bonds. (d) The Sr vacancy with an O–O bond in SrO. (e, f) Two distinct configurations of the Zn vacancy in ZnO, each containing one O–O bond. The small red spheres represent oxygen atoms, while the other spheres denote metal atoms. The oxygen atoms involved in the O–O–O chains are highlighted as yellow spheres. (g) QM/MM energy differences between the reconstructed O–O/O–O–O configurations and the conventional vacancy configuration (set to 0 eV), as a function of charge state for different materials. (h) Defect formation energies of CeO_2_ under O-rich conditions. Energies of *V*_O_, Ce_i_, O_i_, and conventional *V*_Ce_ were adapted from ref 33. The Fermi level is referenced to the valence-band maximum, and the vertical line marks the conduction-band minimum at the experimental band gap.

The energies of the O–O bond and O–O–O chain configurations obtained at the QM/MM level, relative to the conventional vacancy configuration at various charge states, are shown in Figure 3g. At the neutral charge state, for materials with formal oxidation states lower than +4 (namely, MgAl_2_O_4_, ZnO, SrO, and MgO), the energies of reconstructed configurations are higher than those of the corresponding conventional symmetric configurations. In contrast, for SrTiO_3_ and CeO_2_ (oxidation state +4), the configurations with O–O–O chains or O–O bonds lie approximately 2 eV lower than the conventional configurations. This trend is consistent with the MACE predictions and confirms that higher cation oxidation states favor deeper oxygen reconstruction at cation vacancies.

At positive charge states, extra hole localization on the O–O bonds and O–O–O chains stabilizes these reconstructed structures relative to the conventional configurations. The effect is especially pronounced for SrTiO_3_ and CeO_2_, where the neutral reconstructed vacancies are already substantially stabilized; further oxidation lowers the relative energies by up to 3–4 eV. Moreover, in MgAl_2_O_4_, ZnO, and SrO, the neutral O–O-containing vacancies are higher in energy than the corresponding conventional structures. Upon ionization, the added holes localize on the O–O units and stabilize several reconstructed structures, including the +1, +2, and +3 charge states of *V*_Al_ in MgAl_2_O_4_, +1 and +2 charge states of *V*_Sr_ in SrO, and +1 and +2 charge states of *V*_Zn_ in ZnO. Thus, even in lower-oxidation-state oxides, oxygen dimerization can become an effective charge-compensation mechanism when additional holes are introduced.

For negatively charged states, however, a different behavior is observed. In most cases, the energies of these configurations are higher than those of the conventional geometry. CeO_2_ is the only exception, where the reconstructed structure remains slightly lower in energy.

The strong stabilization of the O–O–O configuration has direct consequences for cerium-vacancy thermodynamics in CeO_2_. Under near-equilibrium ambient conditions, oxygen vacancies and Ce^3+^ polarons are expected to dominate the intrinsic defect chemistry of ceria. Cerium vacancies are therefore unlikely to be the dominant native defect under ordinary conditions, but they become relevant under highly oxidizing or nonequilibrium conditions, including irradiation, where cerium vacancies and oxygen interstitials can be generated.^34,35^ The reconstructed Ce–O–O–O–Ce motif gives substantially lower *V*_Ce_ formation energies than the conventional quasi-symmetric vacancy under O-rich conditions in the charge states 0 to +4 (Figure 3h). Thus, using the normal vacancy as the reference can significantly overestimate the thermodynamic cost of forming *V*_Ce_. More broadly, O–O-bond-driven reconstruction is not limited to cation vacancies. A similar stabilization has been found for oxygen interstitials in CeO_2_, where the O–O-bonded configuration (O_2_^2–^) is 1.2 eV lower in energy than the conventional interstitial structure (two holes) in the charge neutral state.^33^

### Generality of Oxygen-Bonded Vacancy Reconstruction across Metal Oxides Validated by Periodic DFT Calculations

Our previous results show that in metal oxides with formal oxidation states of +4 or higher, cation vacancies can undergo substantial oxygen reconstruction, forming O–O bonds or O–O–O chains, significantly lowering the energy of the defective system. The QM/MM approach is currently applied only to a small number of systems because construction of such embedded models requires accurate interatomic potentials and careful parametrization of the QM/MM interface. To test the generality of this finding beyond the representative systems discussed above, we employ the plane-wave DFT code Vienna Ab initio Simulation Package (VASP)^36,37^ to investigate these configurations, restricting the calculations only to the charge-neutral case to avoid introducing artificial image–image interactions of charged defects in periodic supercells. The calculations were performed at the PBEsol +*U* level,^38^ with initial bulk structures and pseudopotentials taken from the Materials Project,^39^ followed by full optimization of the cell, construction of supercells, and defect simulations. Further details are provided in the methodology.

The oxide set covers formal cation oxidation states from +2 to +7. The divalent oxides include BeO, CaO, BaO, and CdO; the trivalent oxides are Al_2_O_3_, Ga_2_O_3_ (two distinct Ga sites), and In_2_O_3_; the tetravalent oxides are TiO_2_, ZrO_2_, HfO_2_, SiO_2_, GeO_2_, SnO_2_, and PbO_2_; the pentavalent oxides include V_2_O_5_, Nb_2_O_5_, Ta_2_O_5_, Sb_2_O_5_, and Bi_2_O_5_; the hexavalent oxides include CrO_3_, MoO_3_, and WO_3_; and the +7 oxidation state is represented by Re_2_O_7_. This series allows the stability of oxygen-bonded vacancy reconstructions to be compared across a wide range of metal–oxygen bonding environments.

The periodic DFT results reproduce the oxidation-state dependence identified by MH and QM/MM. In +2 and +3 oxides, the reconstructed vacancies mainly contain a single O–O bond, as illustrated by BaO and Ga_2_O_3_ in Figure 4a,b. These structures are generally not stabilized relative to the conventional structure. By contrast, most oxides with cation oxidation states of +4 or higher form O–O–O chain configurations with enhanced stability, as shown for TiO_2_, Ta_2_O_5_, CrO_3_, and Re_2_O_7_ in Figure 4c–f. SiO_2_ is the main exception within this higher-oxidation-state group; instead of an O–O–O chain, the silicon vacancy favors two separated O–O bonds, consistent with a previous study by Pacchioni and Ieranò.^40^ The relative energies between the O–O–O (or two O–O) configurations and the conventional vacancy are shown in Figure 4g. GeO_2_ is the only exception in this set for which the reconstructed configuration is not significantly lower in energy.

**Figure 4:**
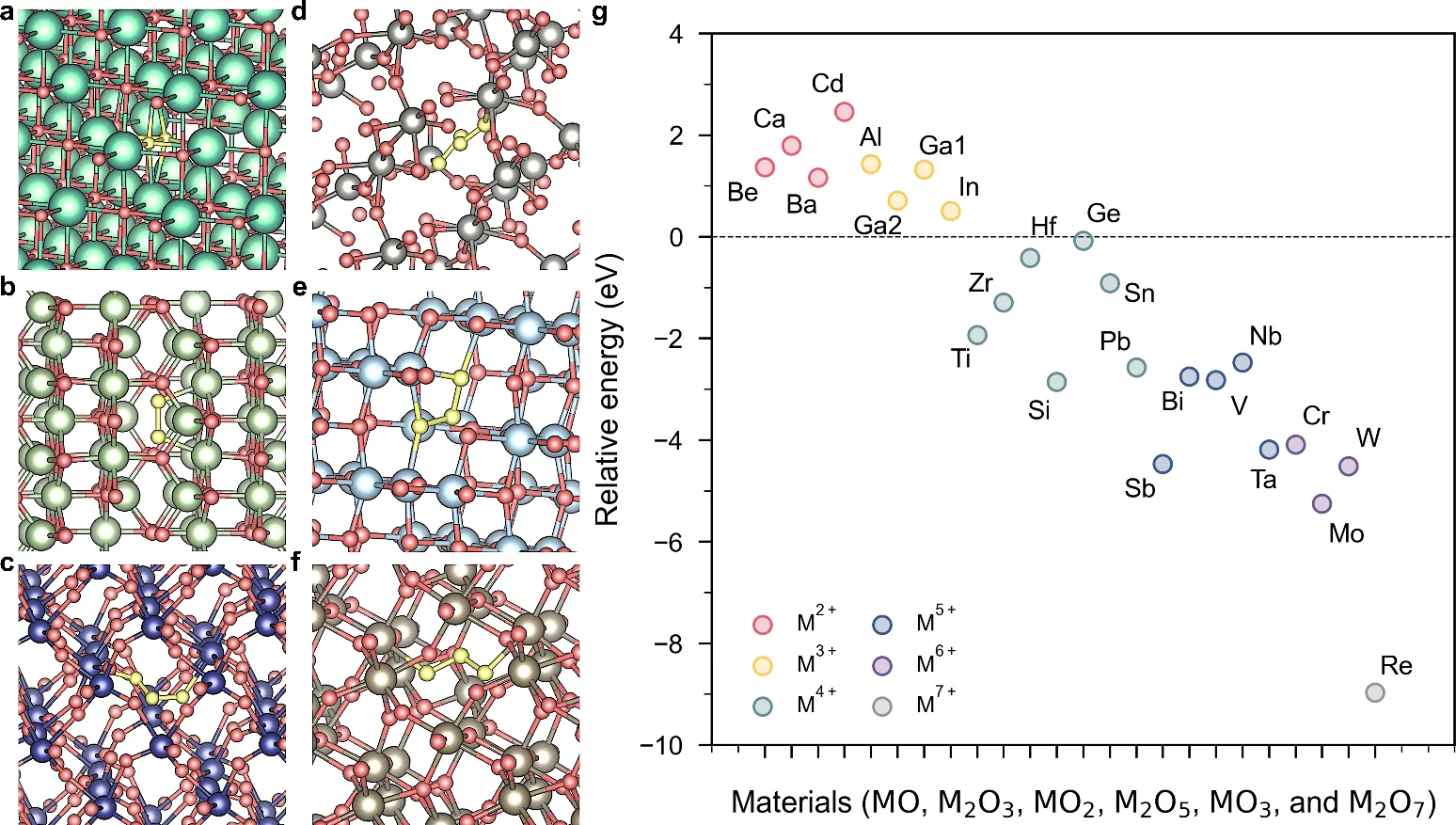
Representative reconstructed oxygen-bonded cation-vacancy configurations in (a) BaO, (b) Ga_2_O_3_, (c) TiO_2_, (d) Ta_2_O_5_, (e) CrO_3_, and (f) Re_2_O7. Other materials are provided in Figures S17–S19 in Supporting Information. (g) Relative energies of the reconstructed O–O–O or two O–O defect configurations with respect to the conventional cation vacancy configuration in the charge-neutral state for various metal oxides. The energy of the conventional configuration is set to 0 eV for each material. The small red spheres represent oxygen atoms, while the other spheres denote metal atoms. The oxygen atoms involved in the O–O–O chains or O–O bonds are highlighted as yellow spheres.

The trend therefore extends beyond the specific systems used in the QM/MM analysis and supports a general chemical picture. For the materials discussed in this work, a common feature is that the holes generated by cation removal localize predominantly on the undercoordinated oxygen atoms surrounding the vacancy. In high-oxidation-state oxides, these oxygen-centered holes can be further stabilized through reconstruction of the local oxygen network and formation of O–O or O–O–O motifs. By contrast, in lower-valent oxides, the isolated-hole configurations generally remain energetically preferred.

Exceptions arise when other metal sites in oxides can accommodate the holes generated by cation removal. In UO_2_, for example, the VBM is mainly composed of U 5f states, and the excess holes introduced by the uranium vacancy preferentially localize on the neighboring U sites rather than on oxygen, forming U^5+^ polarons.^41–43^ This metal-centered charge-compensation mechanism suppresses the driving force for O–O and O–O–O formation, despite UO_2_ adopting the same cubic fluorite structure as CeO_2_. A similar situation also occurs in hematite (α-Fe_2_O_3_), FeO, NiO, and MnO, where the states near the VBM have mixed O 2p and metal 3d character, and hole polarons have been reported to localize on metal sites rather than oxygen.^44–46^ We confirmed this behavior by periodic DFT calculations for both UO_2_ and FeO. Therefore, the formation of O–O and O–O–O motifs can also be viewed as a consequence of the inability of the metal sublattice to accommodate the holes generated by cation removal.

Our approach is applicable not only to binary metal oxides but also to mixed-metal oxides, as demonstrated here for SrTiO_3_ and MgAl_2_O_4_. It can also be extended to other complex oxides, such as CaZrTi_2_O_7_ and In_2_ZnO_4_, as well as to other compounds, including CH_3_NH_3_PbI_3_ and CsPbBr_3_. As demonstrated in previous studies,^13^ in such chemically and structurally complex systems, the defect configurational space can become substantially larger, making global structure prediction particularly important for identifying relevant low-energy defect configurations.

Hydrogen may also influence the stability of O–O or O–O–O motifs by modifying the local charge-compensation mechanism, for example through hydroxyl formation or interaction with holes. However, because the present study focuses on intrinsic cation-vacancy-induced reconstruction, hydrogen impurities are not considered. O–O–O species have previously been identified in transition-metal clusters and other molecular systems, including cases involving metals in relatively low oxidation states.^47,48^ Our results show that analogous O–O–O units can also form within defective crystalline metal oxides, where the configurations are stabilized by the host oxide lattice with metals in high oxidation states.

The strength and nature of the metal–oxygen (M–O) electrostatic interactions are important factors in determining the defect structures. This is also a multiminimum problem arising from the possible bonding rearrangements around a defect. For lower oxidation states, the energetic cost associated with disrupting the surrounding M–O electrostatic interactions upon cation removal is relatively small. Moreover, in many oxides such as MgO and SrO, the highly symmetric and compact lattice strongly constrains the local geometry, providing relatively little structural freedom for substantial reconstruction. In contrast, removal of a highly charged cation produces a much larger local electronic and electrostatic perturbation. The system can lower its energy by reorganizing the local oxygen network, including through the formation of new O–O bonds. Whether such reconstruction occurs therefore depends on the competition between maintaining the original M–O coordination and forming alternative O–O bonding configurations, together with the structural flexibility of the host lattice.

Finally, the thermodynamic and experimental conditions governing the formation of cation vacancies should be distinguished from the energetic ordering of their possible reconstructed configurations. In the high-oxidation-state oxides for which O–O reconstruction is favored, the formation of a cation vacancy can itself be energetically costly because removal of a highly charged cation disrupts strong electrostatic interactions. Consequently, the formation of cation vacancies may be less favorable than other intrinsic defects. However, cation vacancies are generally stabilized under oxygen-rich or metal-poor conditions. Their concentrations can also be strongly affected by deviation from stoichiometry and extrinsic doping, as well as irradiation or energetic-particle bombardment under nonequilibrium conditions. The present work therefore focuses on the structures adopted once such vacancies are formed and shows that O–O dimerization can substantially stabilize the defect relative to the conventional vacancy configuration.

## Summary and Conclusions

This work establishes O–O dimerization as a general charge-compensation pathway for cation vacancies in metal oxides. Minima hopping global structure searches, hybrid QM/MM calculations, and periodic DFT consistently show that in the charge-neutral state, oxides with formal cation oxidation states of +3 or lower generally do not favor O–O-bonded vacancy reconstructions, whereas oxides with cation oxidation states of +4 or higher can stabilize an O–O–O chain or two O–O bonds, often by several electronvolts relative to the conventional quasi-symmetric vacancy. Each isolated O–O bond or O–O–O chain can accommodate at most one additional hole and stabilize such configurations in positive charge states, even in oxides with formal cation oxidation states of +2 and +3. These findings show that assuming conventional defect geometries could miss the energetically preferred defect structures in metal oxides. Predictive defect modeling in solid-state materials should therefore move beyond local relaxation of a single lattice-derived defect model and include global searches for hidden reconstructed configurations.

## Methodology

### Minima Hopping Structure Prediction

The minima hopping (MH) algorithm^49^ was used as a global structure-search procedure for exploring the configurational landscape of charge-neutral cation vacancies in metal oxides. The calculations were performed using a Python implementation^24^ of MH coupled to the Atomic Simulation Environment.^50^ For each oxide, periodic supercells containing a cation vacancy were generated as starting geometries by removing one target cation from the optimized bulk lattice structure. MH alternates short molecular dynamics escape trajectories with local geometry optimization to sample the potential energy surface, enabling efficient sampling of the potential energy surface and identification of distinct local minima, including highly reconstructed defect motifs. Energies and forces were evaluated using MACE-OMAT-0,^51,52^ a foundation machine-learned interatomic potential (MLIP) covering a broad chemical space of 89 elements. This broad elemental coverage makes it suitable as a first-stage structure discovery model for comparing defect reconstructions across diverse metal oxides, although final energetic ordering was validated by higher-level DFT calculations.

During the MH searches, the cell parameters were fixed. Boundary constraints were applied to preserve the bulk-like environment surrounding the defect. Atoms with fractional coordinates satisfying 0.0 ≤ *f*_*i*_ ≤ 0.1 or 0.9 ≤ *f*_*i*_ ≤ 1.0, where *i* = *x*, *y*, *z*, were fixed in all directions. All other atoms were allowed to relax. This setup maintained a rigid outer framework while allowing the inner defective region to undergo local rearrangement.

During the MH sampling, each relaxed structure was compared with previously visited minima in the database using an overlap-matrix structural fingerprint.^53^ Structures with equivalent fingerprints within the chosen similarity threshold were assigned to the same minimum and counted only once. The standard MH feedback mechanism was used to adapt the kinetic energy of the molecular-dynamics escape step. Revisiting the current or a previously identified minimum increased the kinetic energy to facilitate escape from the explored region, whereas locating a new minimum reduced it. This reduction favors low-barrier transitions, which, according to the Bell–Evans–Polanyi principle, are more likely on average to access low-energy minima. Configurations in which the vacancy migrated away from the originally selected cation site, for example, through displacement of a neighboring cation into the vacancy position, were also considered as duplicates and excluded. The final set of unique structures was classified according to the local oxygen connectivity around the vacancy with a cutoff radius of 1.8 Å, such as no O–O bond, one isolated O–O bond, two isolated O–O bonds, one O–O–O chain, and other combined patterns. Representative low-energy structures obtained from the MH searches were then selected for higher-level QM/MM and periodic DFT calculations.

### Hybrid QM/MM Embedded-Cluster Calculations

For the hybrid quantum mechanical/molecular mechanical (QM/MM) embedded-cluster approach, the Python-based version of the ChemShell package^32,54,55^ was employed. The systems selected for QM/MM calculations were SrTiO_3_, CeO_2_, MgAl_2_O_4_, ZnO, SrO, and MgO. For CeO_2_,^22^ ZnO,^56^ and SrO,^16^ we have reported the QM/MM settings, and one can refer to our previous work. Each embedded-cluster model consisted of a metal centered QM cluster embedded in a large MM region. The number of QM and MM atoms are shown in Table S1 in the Supporting Information. The QM cluster is treated quantum mechanically by a hybrid DFT functional and the surrounding region is treated at the MM level of theory using polarizable shell-model interatomic potentials. A cationic interface region between the QM and MM regions serves as the buffer layer to minimize the mismatch of the QM and MM levels of theory. Here, we use specially designed local embedding pseudopotentials on the cationic sites in the form of a linear combination of three Gaussian functions as in eq 1 and with the fitted parameters for the cations in atomic units as given in Table S2 in Supporting Information.

$$r^{2}U_{p}\left(r\right)=A_{1}re^{-Z_{1}r^{2}}+A_{2}r^{2}e^{-Z_{2}r^{2}}+A_{3}r^{2}e^{-Z_{3}r^{2}}$$ 1

For the QM part of the calculations, we used triple-ζ plus polarization Gaussian basis sets^57^ on O, Sr, Ti, Ce, Mg, and Al including 28-electron effective core potentials (ECPs) on Sr^58^ and Ce, as implemented in the NWChem^59^ software. To reduce the computational cost, we have removed the *f* function from the oxygen basis set and some of the highly diffuse functions from the cation basis sets, which do not contribute to ionic bonding. For electron exchange and correlation, the BB1K^60^ hybrid meta-GGA functional with 42% exact exchange was used for the results shown in the main text. The PBE0 ^61,62^ functional with 25% exact exchange was also used for some systems and the two functionals gave consistent trends. For the MM part of the simulation we have used pairwise shell-model interatomic potentials that have been fitted to reproduce the structural, elastic, dynamical, and dielectric properties of the materials. Details on the force fields can be found in ref 22 for CeO_2_, ref 16 for SrO, ref 63 for ZnO, and refs 64 and 65 for MgO. For SrTiO_3_, we adopted the potential from ref 66 with slight modifications as detailed in Tables S3 and S4 in Supporting Information. For MgAl_2_O_4_, the potentials were taken from refs 67 and 68 and slightly modified as similar as SrTiO_3_ and detailed in Table S5 in Supporting Information. The General Utility Lattice Package (GULP)^69,70^ is employed for the MM part of the calculation.

### Periodic DFT Calculations

We carried out plane-wave DFT calculations using VASP^36,37^ . For the exchange and correlation term, the Perdew–Burke–Ernzerhof (PBE)^71^ functional as revised for solids (PBEsol^38^) was used. The electronic wave functions were expanded in a plane wave basis set with an energy cutoff of 650 eV. Self-consistent DFT energies were converged to 10^–6^ eV, and geometry optimization was deemed converged when the residual forces on the atoms were smaller than 1 × 10^–3^ eV/Å. The unit cells of the materials and pseudopotentials of the elements used in all calculations were taken from the Materials Project.^39^ The unit cells were fully optimized; the *k*-point meshes were chosen such that *a*_*x*_ × *k*_*x*_ ∼ 30 (*x* = 1, 2, 3). For materials with excess d electrons, the DFT+*U* ^72^ was applied with *U*_eff_ = *U* – *J* = 5 eV and a fixed *J* value of 1.0 eV. To model the cation vacancies, supercells with each lattice dimension expanded to ∼20 Å were used, including more than 200 atoms. The k-point mesh 2 × 2 × 2 was used in the supercell calculations.

## Supplementary Material



## Notes

Density of configurations (DOC) from minima hopping algorithm calculations for MgAl_2_O_4_, MnO, Mn_2_O_3_, Mn_3_O_4_, MnO_2_, SrO, SrTiO_3_, TiO_2_, and MgO; the QM/MM details, including cluster sizes, pseudopotential parameters, MM interatomic potentials for SrTiO_3_ and MgAl_2_O_4_ and spin density distributions for SrO, MgO, and ZnO; and periodic DFT results for configurations containing O–O bonds or O–O–O chains in BeO, CaO, CdO, Al_2_O_3_, Ga_2_O_3_, In_2_O_3_, ZrO_2_, HfO_2_, SiO_2_, GeO_2_, SnO_2_, PbO_2_, Sb_2_O_5_, Bi_2_O_5_, V_2_O_5_, Nb_2_O_5_, MoO_3_, and WO_3_ (PDF)
