# Supplementary material for: Cation Vacancies with and without O–O Dimerization: Identifying Reconstructed Defect Configurations in Metal Oxides
Source: J Am Chem Soc. 2026 Oct 7;148(41):44973–83. doi: 10.1021/jacs.6c15378 (PMC13693452; doi:10.1021/jacs.6c15378)
Supplement: Supplementary file 1 [file ja6c15378_si_001.pdf]

Supplementary Information

Cation Vacancies with and without O–O Dimerization: Identifying Reconstructed Defect Configurations in Metal Oxides

Taifeng Liu[1, 2#,*], Xingfan Zhang[2, 3#], Jamal Abdul Nasir[2], Qing Hou[2, 4], Liam Morgan[2, 5], Jingcheng Guan[2], Thomas Durrant[5], You Lu[5], Thomas W. Keal[5], John Buckeridge[6*], Scott M. Woodley[2], Stefan Goedecker[3], C. Richard A. Catlow[2,7*], Alexey A. Sokol[2*]

1. National & Local Joint Engineering Research Center for Applied Technology of Hybrid Nanomaterials, Henan University, Kaifeng 475004, P. R. China.
2. Kathleen Lonsdale Materials Chemistry, Department of Chemistry, University College London, 20 Gordon Street, London, WC1H0AJ, United Kingdom.
3. Department of Physics, University of Basel, Klingelbergstrasse 82, CH-4056 Basel, Switzerland.
4. Institute of Photonic Chips, University of Shanghai for Science and Technology, 580 Jungong Road, Shanghai 200093, China.
5. Scientific Computing Department, STFC Daresbury Laboratory, Warrington WA4 4AD, Cheshire, United Kingdom.
6. School of Engineering, London South Bank University, 103 Borough Road, London SE1 OAA, United Kingdom.
7. School of Chemistry, Cardiff University, Park Place, Cardiff, CF10 1AT, United Kingdom.

Corresponding authors: T. Liu: tfliu@vip.henu.edu.cn; J. Buckeridge: j.buckeridge@lsbu.ac.uk; A. Sokol: a.sokol@ucl.ac.uk; R. Catlow: c.r.a.catlow@ucl.ac.uk

#T. Liu and X. Zhang contributed equally to this work.

1. Density of configurations (DOC) for $MgAl_2O_4$, MnO, Mn_2O_3, Mn_3O_4, MnO_2, SrO, $SrTiO_3$, TiO_2, and MgO)

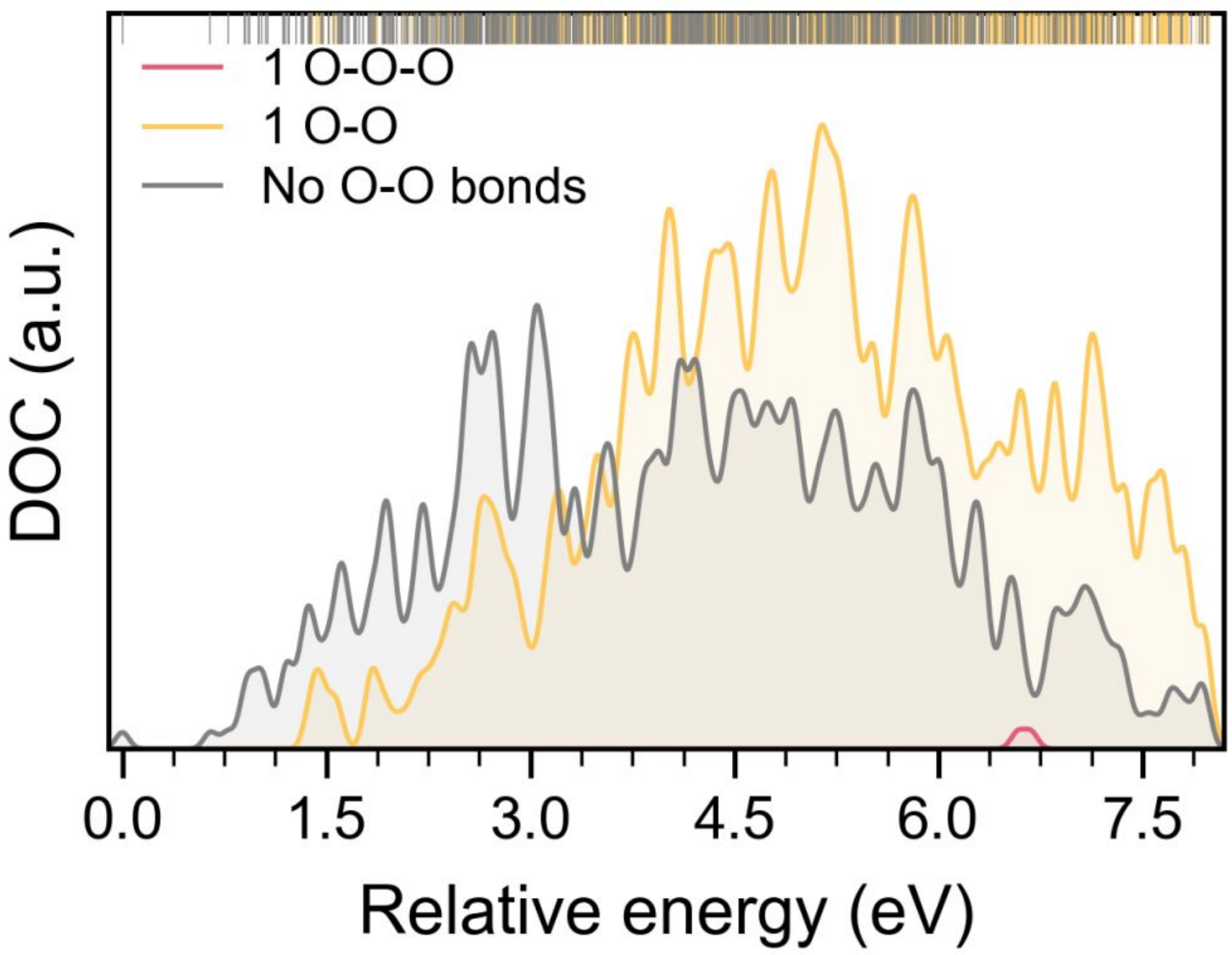

Figure S1. Density of configurations (DOC) as a function of relative energy sampled by the MH simulations for Al vacancies in $MgAl_2O_4$.

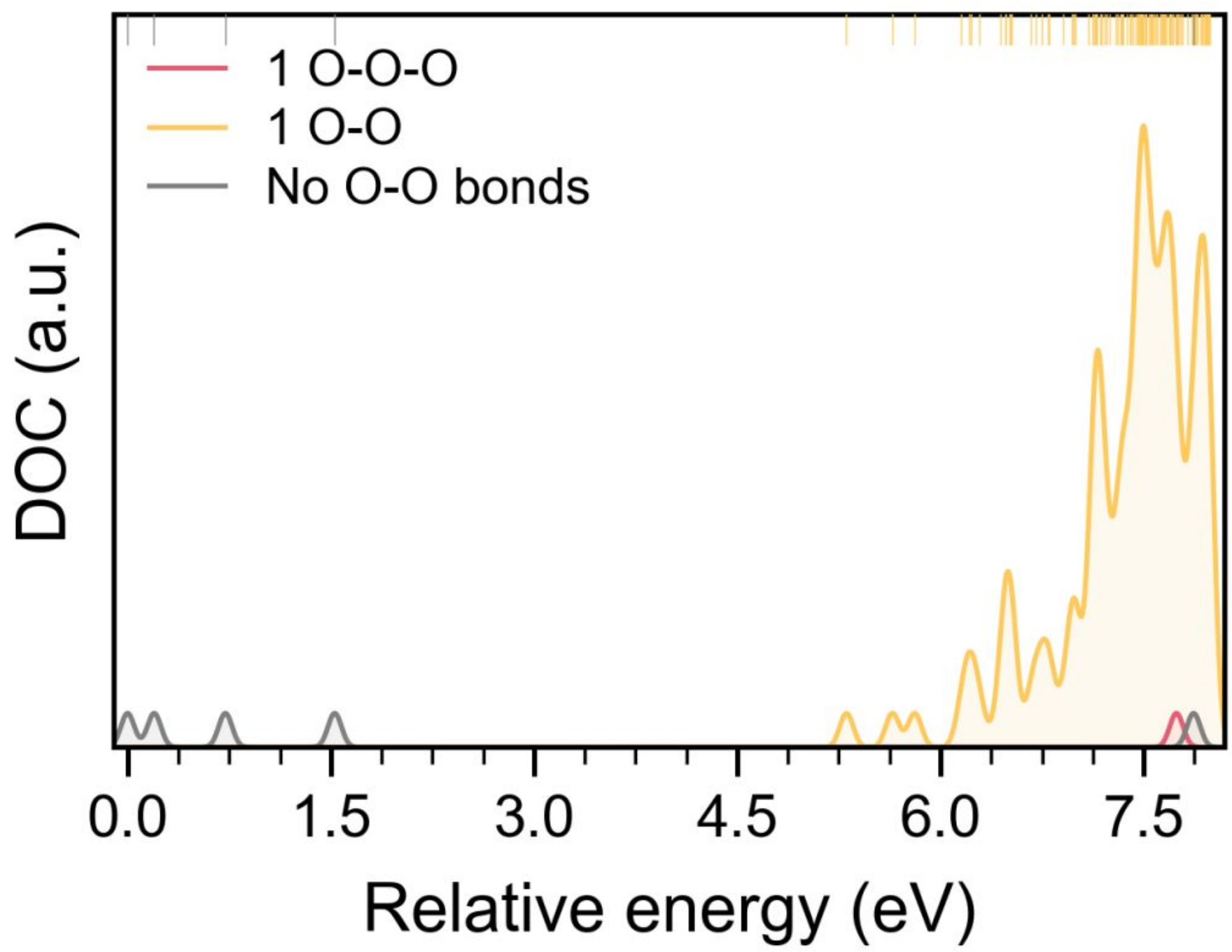

Figure S2. Density of configurations (DOC) as a function of relative energy sampled by the MH simulations for Mg vacancies in $MgAl_2O_4$.

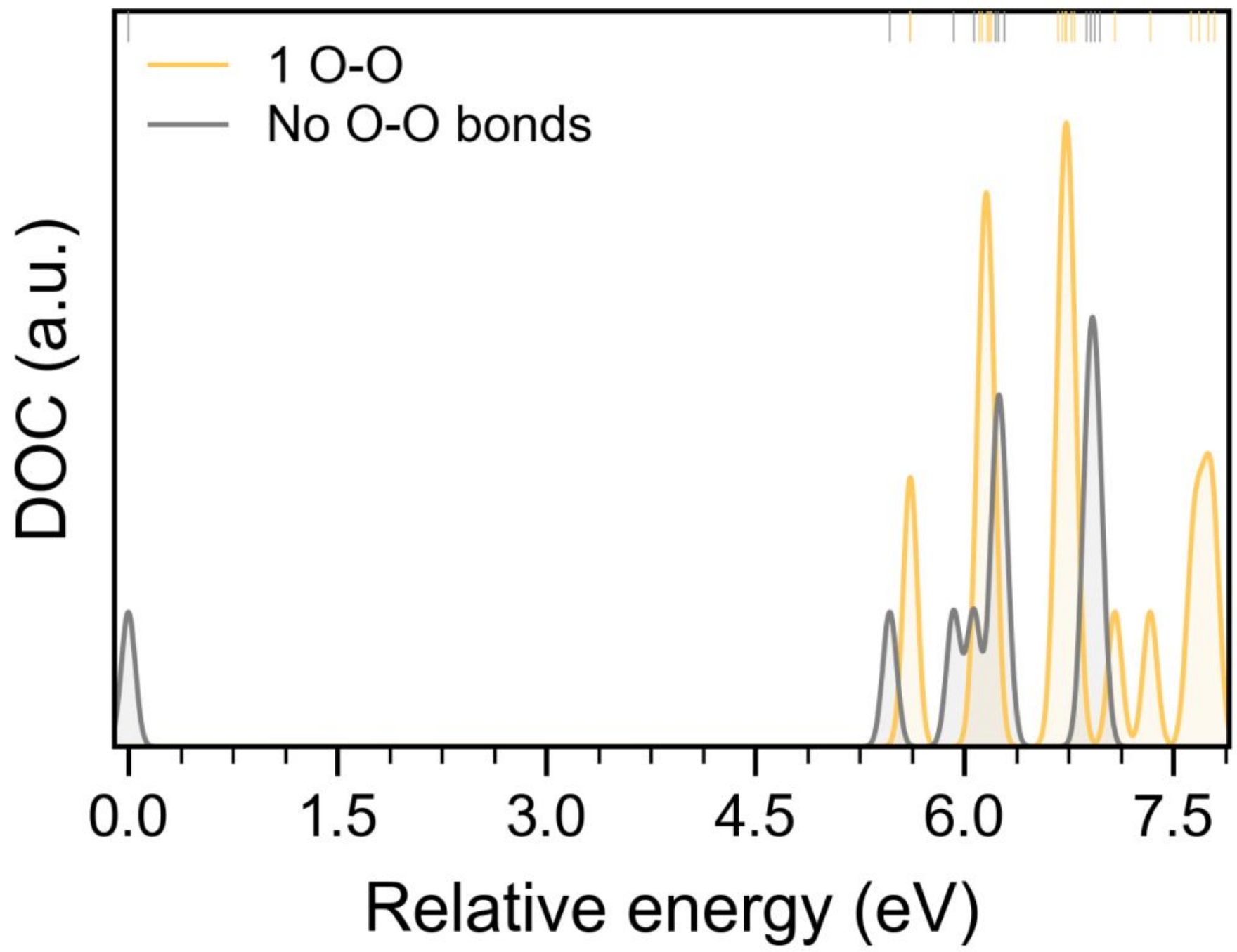

Figure S3. Density of configurations (DOC) as a function of relative energy sampled by the MH simulations for Mn vacancies in MnO.

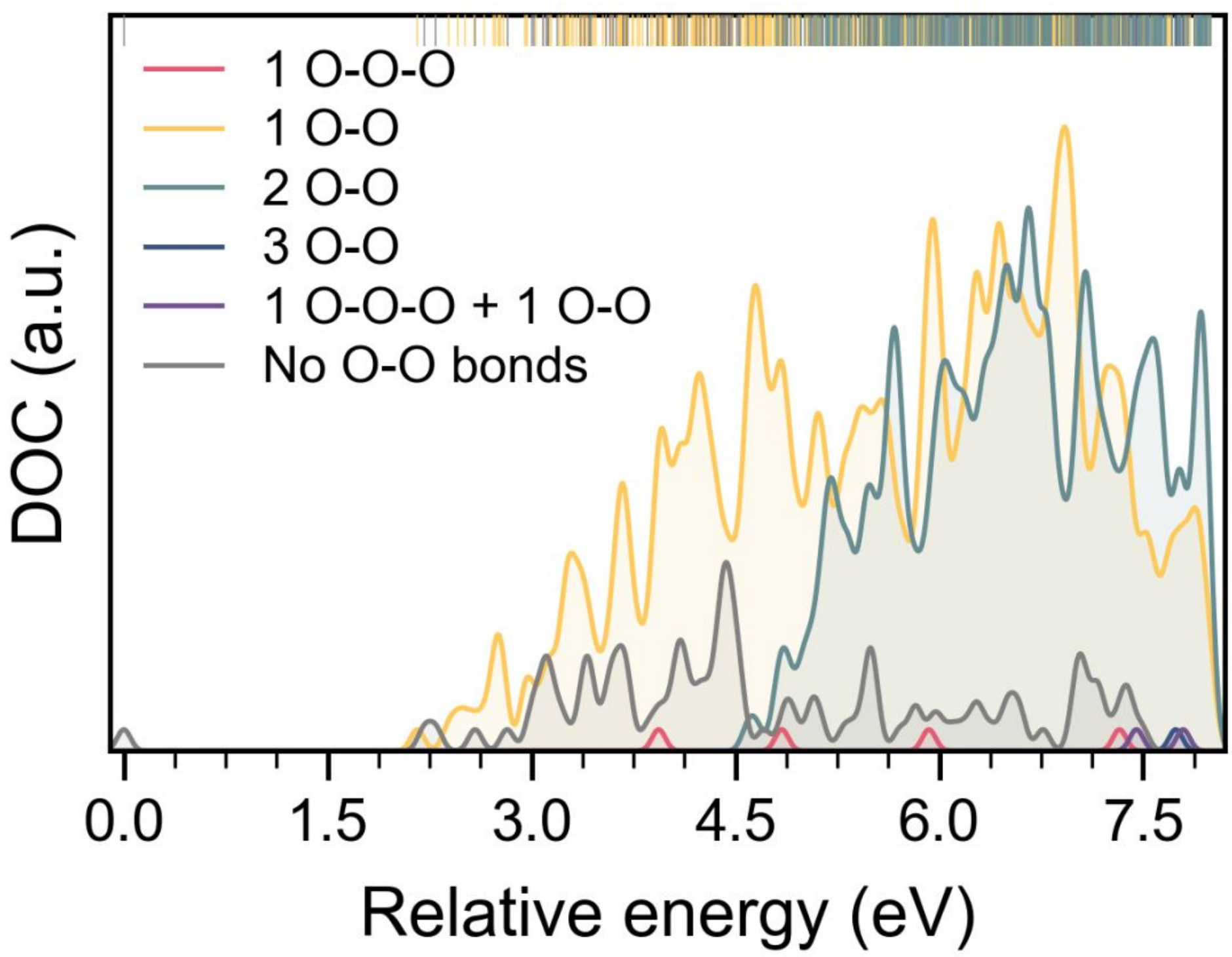

Figure S4. Density of configurations (DOC) as a function of relative energy sampled by the MH simulations for Mn vacancies in Mn_2O_3.

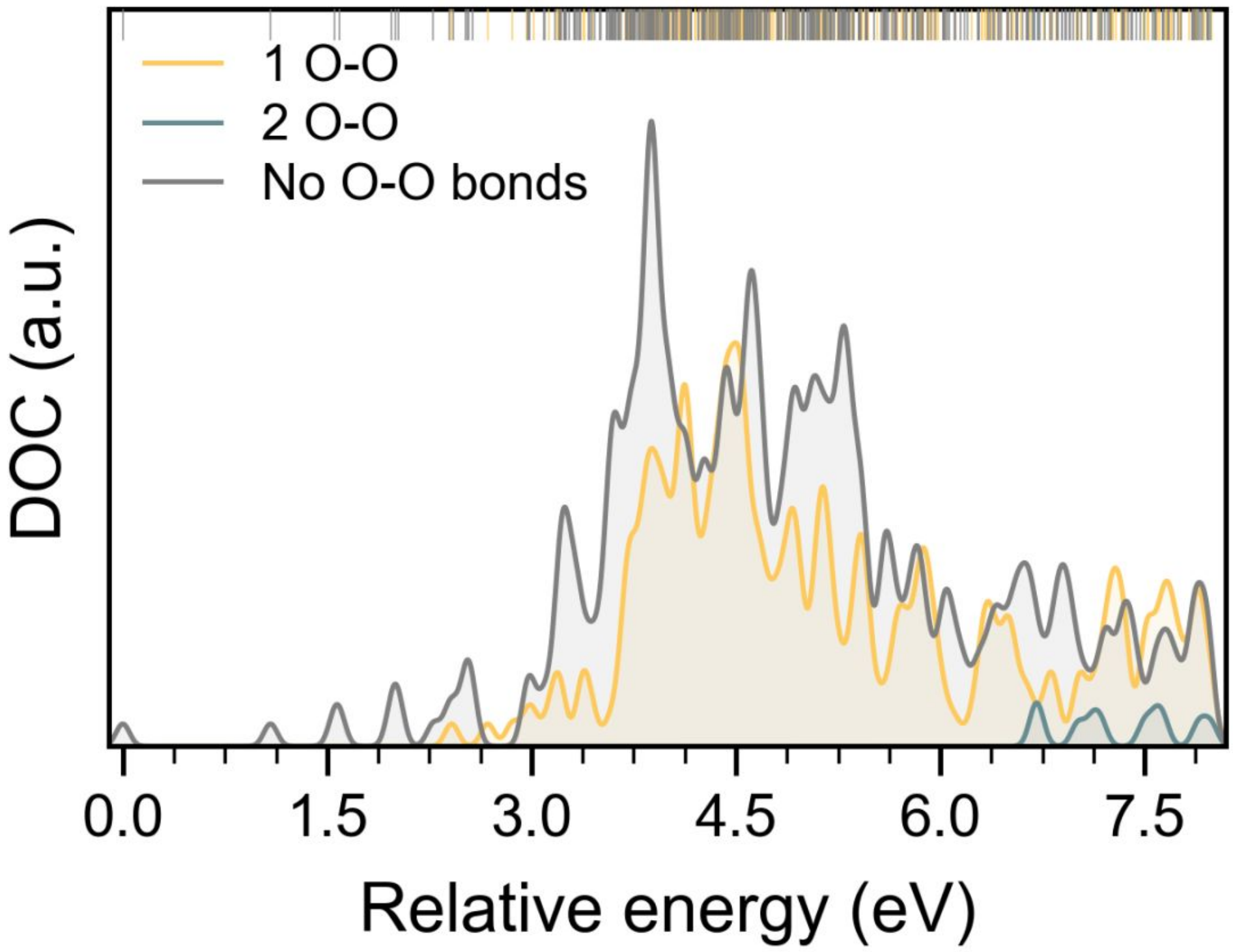

Figure S5. Density of configurations (DOC) as a function of relative energy sampled by the MH simulations for Mn vacancies in Mn_3O_4.

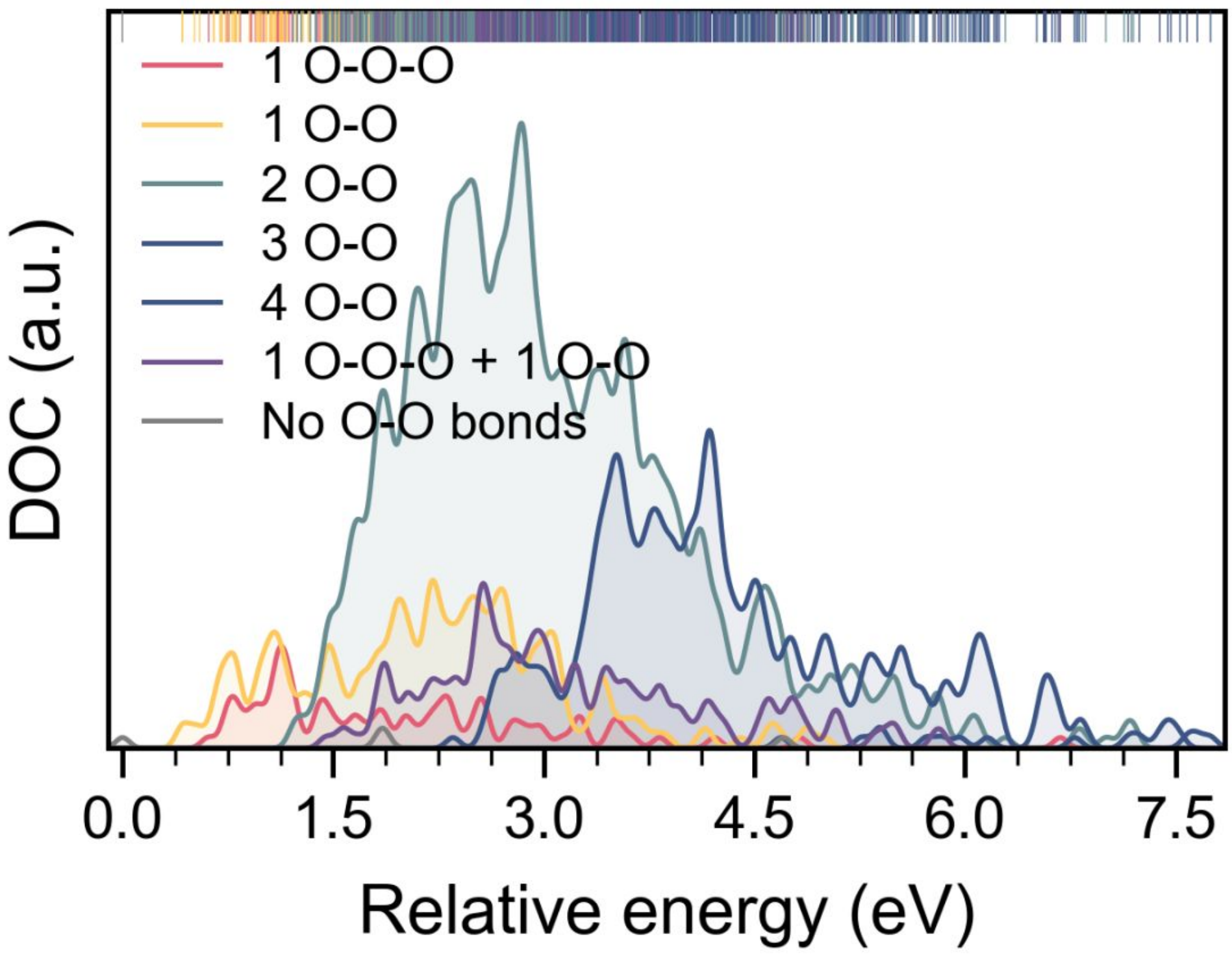

Figure S6. Density of configurations (DOC) as a function of relative energy sampled by the MH simulations for Mn vacancies in MnO_2.

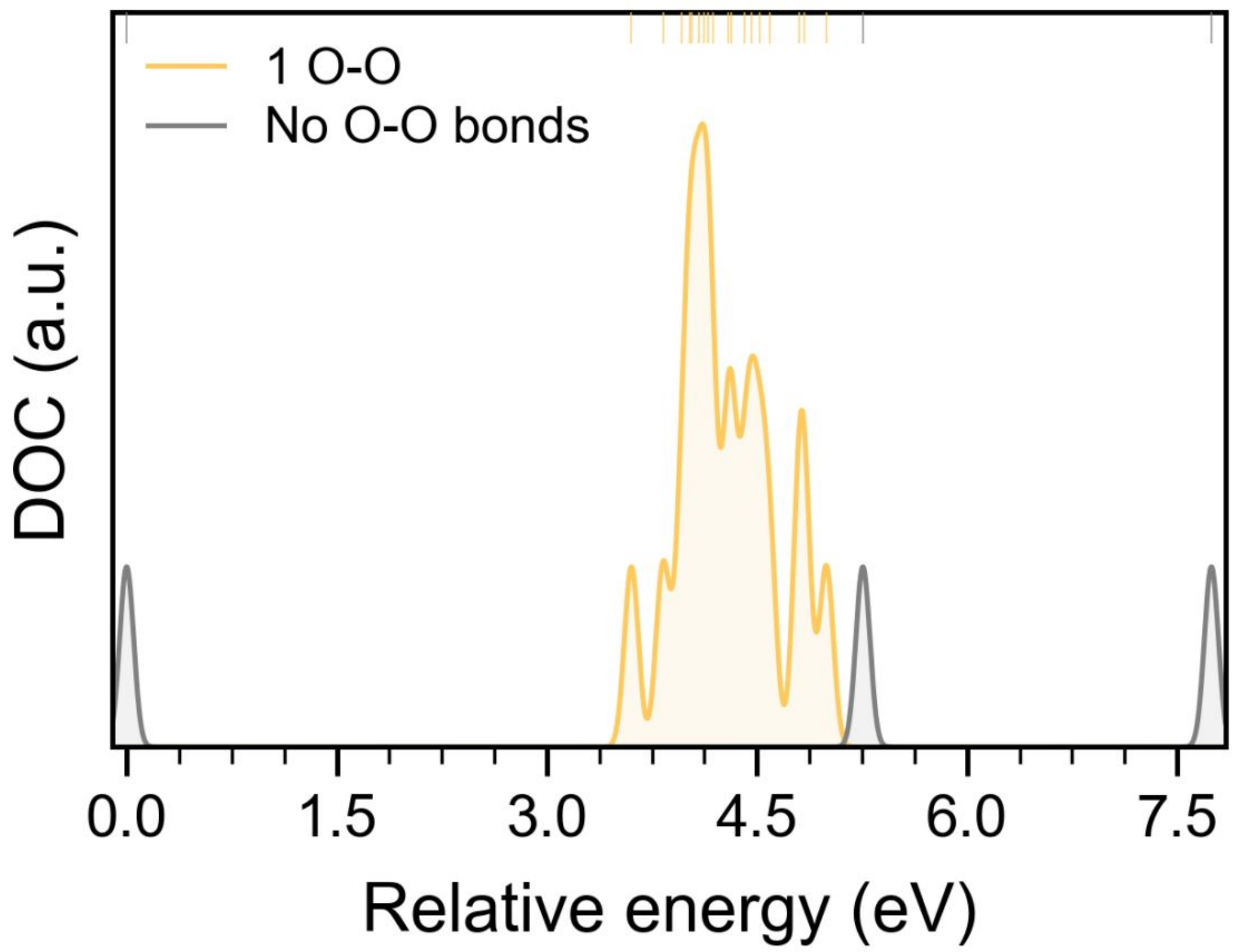

Figure S7. Density of configurations (DOC) as a function of relative energy sampled by the MH simulations for Sr vacancies in SrO.

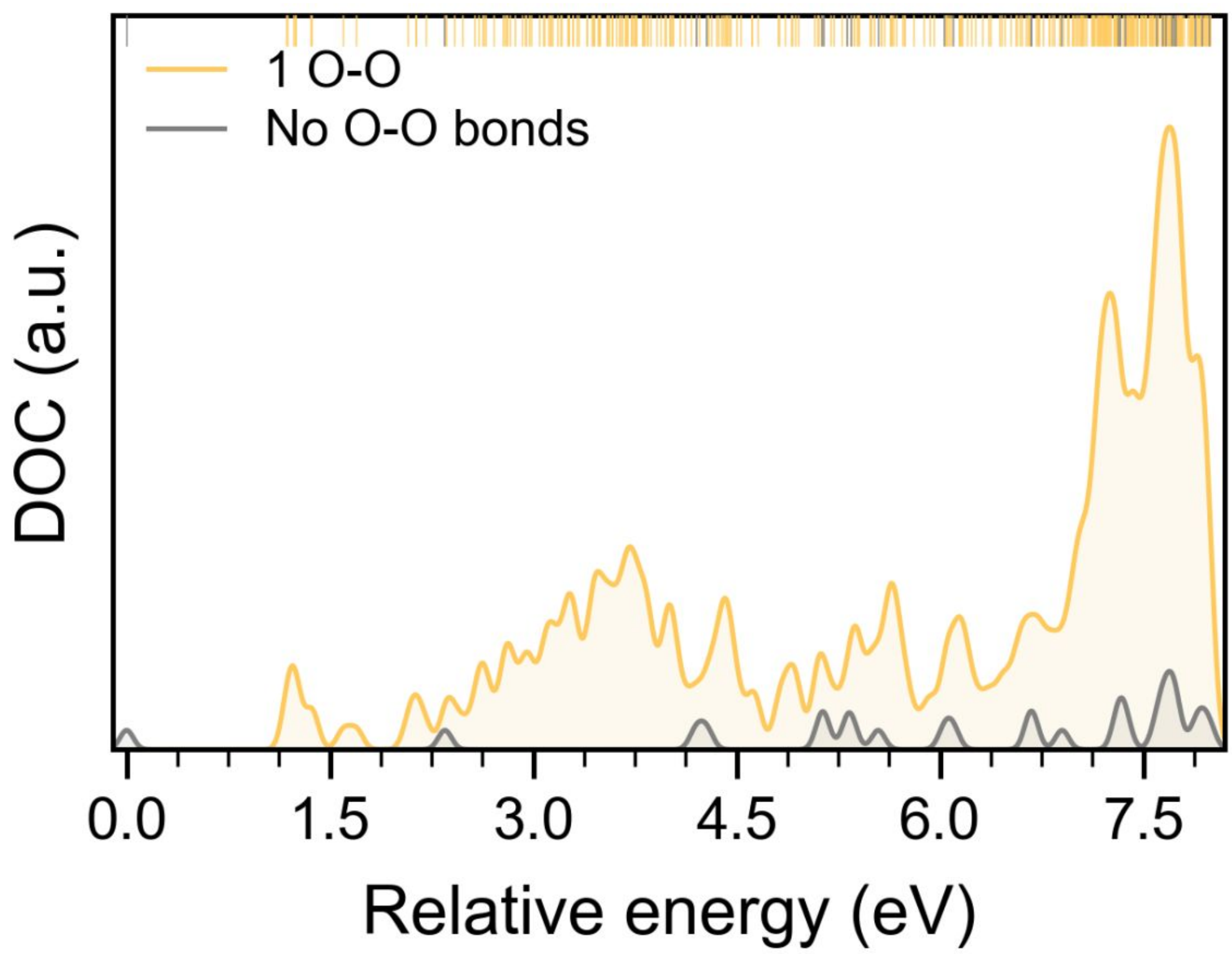

Figure S8. Density of configurations (DOC) as a function of relative energy sampled by the MH simulations for Sr vacancies in $SrTiO_3$.

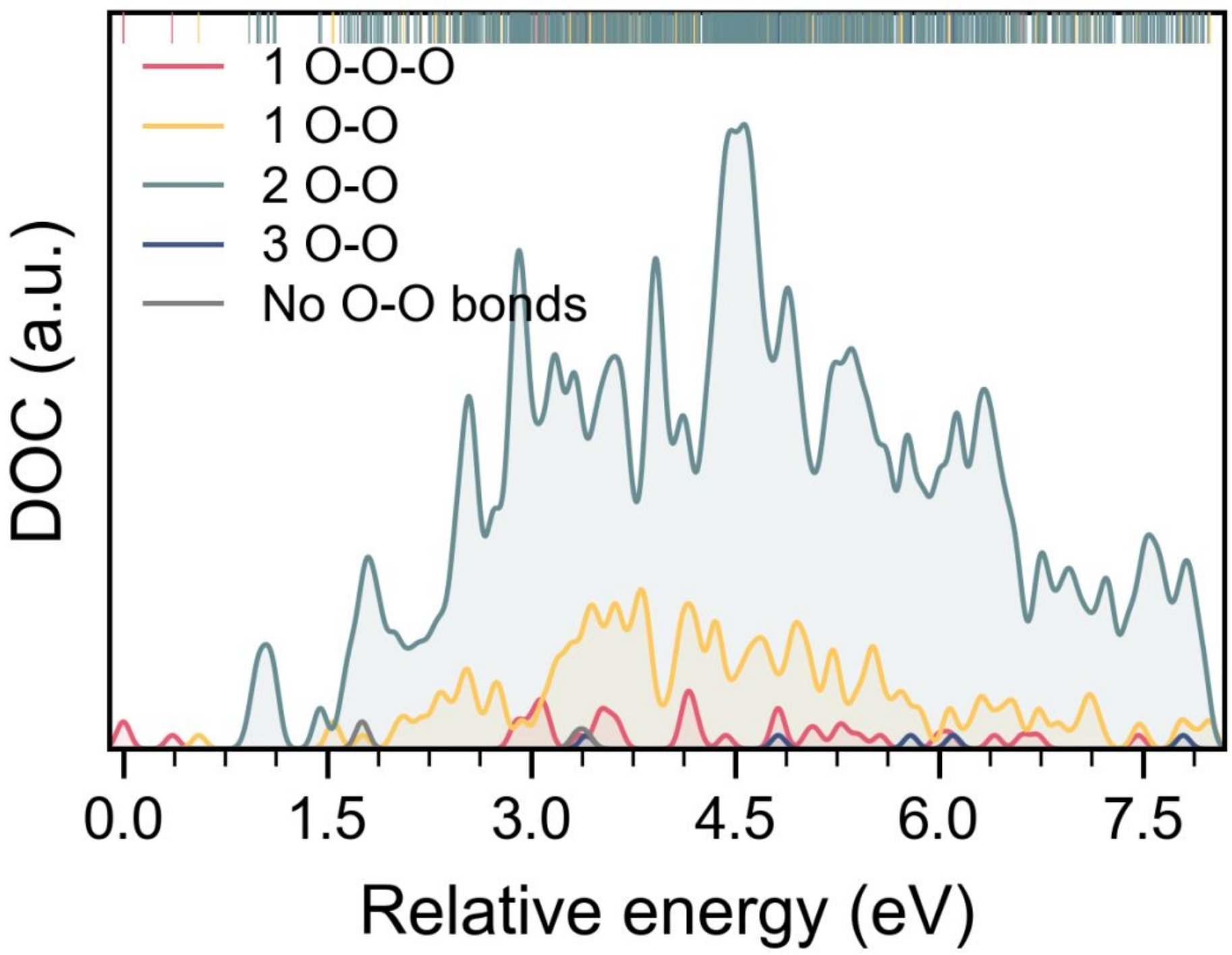

Figure S9. Density of configurations (DOC) as a function of relative energy sampled by the MH simulations for Ti vacancies in $SrTiO_3$.

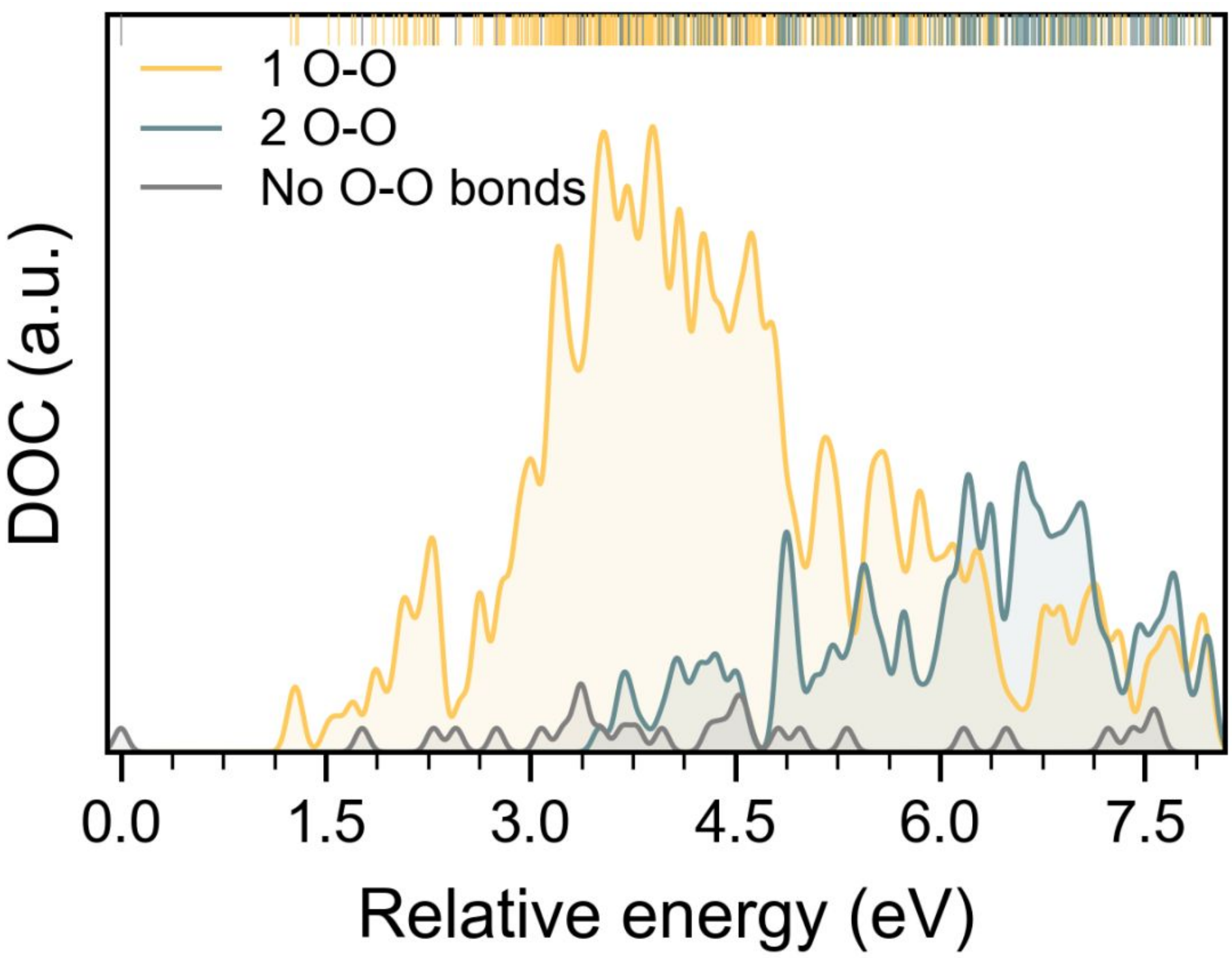

Figure S10. Density of configurations (DOC) as a function of relative energy sampled by the MH simulations for Ti vacancies in anatase TiO_2.

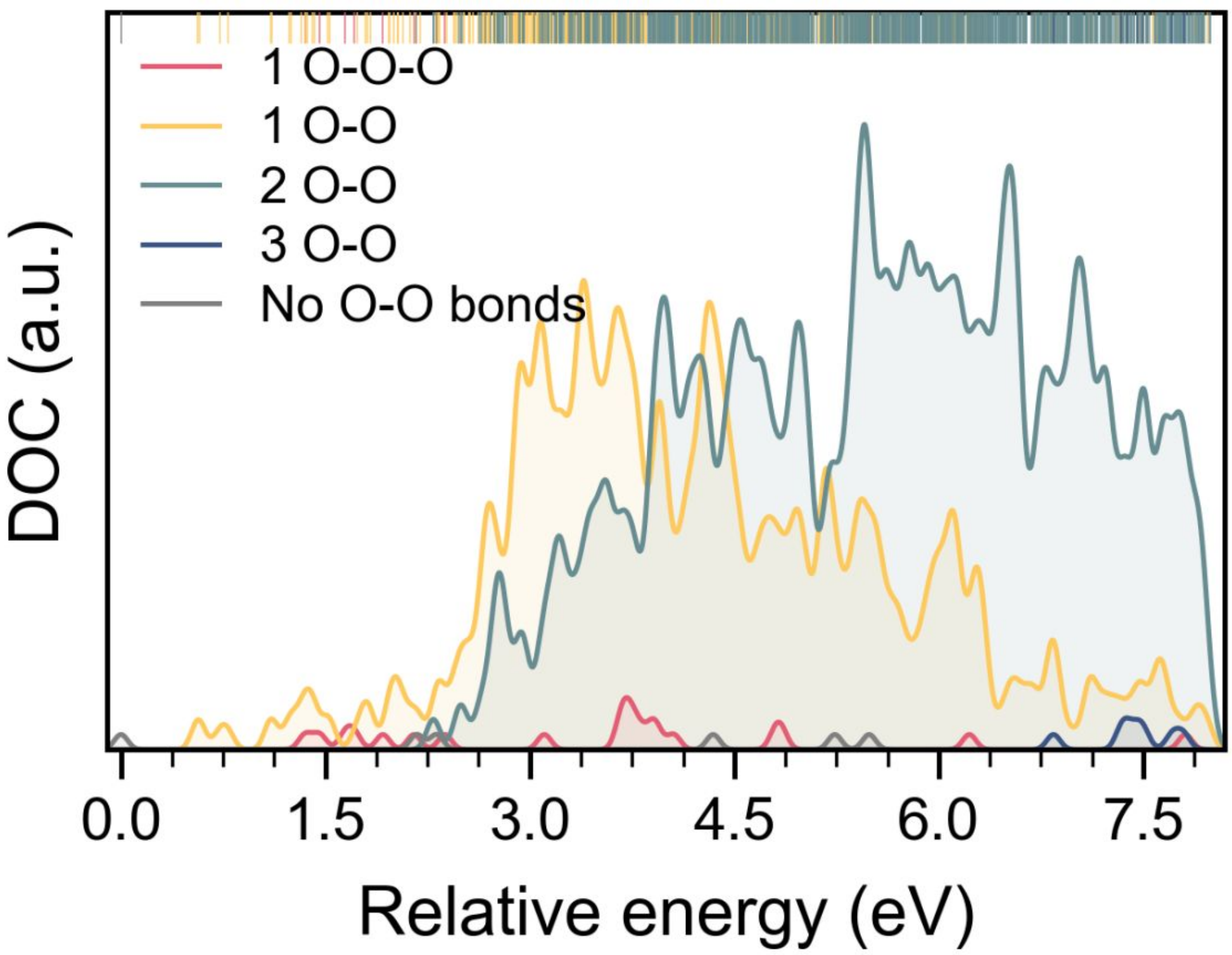

Figure S11. Density of configurations (DOC) as a function of relative energy sampled by the MH simulations for Ti vacancies in rutile TiO_2.

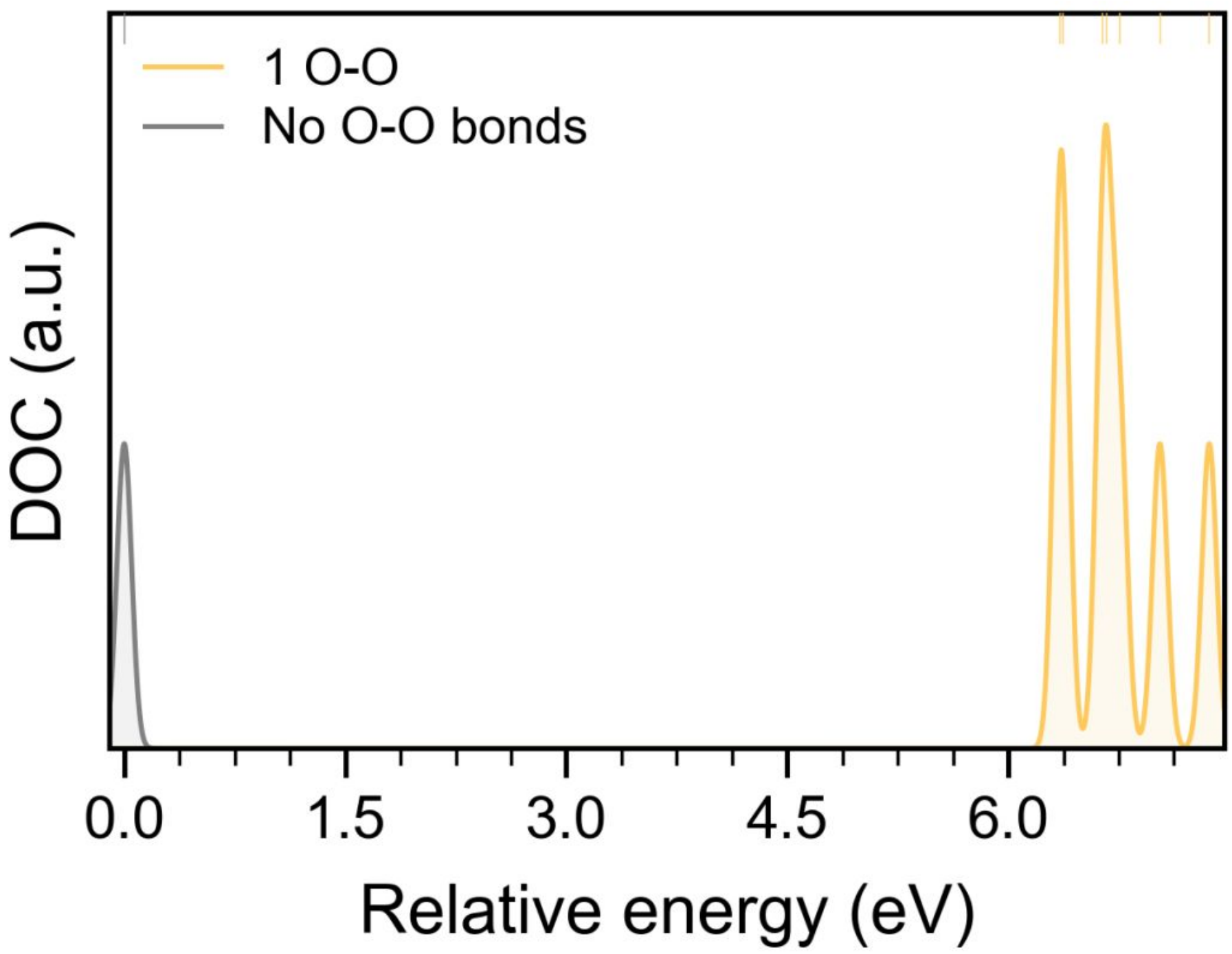

Figure S12. Density of configurations (DOC) as a function of relative energy sampled by the MH simulations for Mg vacancies in rutile MgO.

2. Details of hybrid quantum mechanical/molecular mechanical (QM/MM) embedded-cluster approach

2.1 The size of cluster

For the cluster size, in all cases we employed a metal-centered QM cluster embedded within a larger MM cluster. The number of atoms in each cluster is listed in Table S1.

Table S1. The details of the cluster in the QM/MM setting.

Material	QM-size	Total size
$SrTiO_3$	149-Ti centered	10891
CeO_2	110	9898
$MgAl_2O_4$	95-Al centered	13151
ZnO	85	10545
MgO	55	4600

2.2 Pseudopotential parameters

The specially designed local embedding pseudopotentials on the cationic sites are expressed as a linear combination of three Gaussian functions, as shown in Equation (1) in the main text. The fitted parameters for the cations, in atomic units, are provided in Table S2.

Table S2. Embedding cation pseudopotential parameters in atomic units [see Eq. 1 in the main text]

Elements	i	A_i	Z_i
	1	-52.9247	20.9445
Sr	2	21.3965	1.26349
	3	2.07299	0.675973
Ti	1	-88.1067	7.35
	2	48.9402	3.2652
	3	18.10	1.35
Ce	1	-49.817	24.9589
	2	60.247	2.85901

	3	0.295877	0.25855
Mg	1	14.67600000	1.73200000
	2	5.17570000	1.11500000
	3	-1.81600000	1.20300000
Al	1	20.28390000	2.19830000
	2	8.55710000	1.59770000
	3	-3.51980000	1.97670000

2.3 Interatomic potentials of MM part for $SrTiO_3$ and $MgAl_2O_4$.

A new shell-model interatomic potential for $SrTiO_3$ was developed using the GULP code. To describe the relative stability of the cubic, tetragonal, and rhombohedral phases, with particular emphasis on reproducing the rhombohedral ground-state phase, potential set C from Ref.[1] was slightly modified. The resulting potential parameters are listed in Table S3. The new potential accurately reproduces the lattice parameters and relative phase stability, as shown in Table S4, where comparisons with DFT[2] and experimental results are also provided. Similarly, the potential parameters of $MgAl_2O_4$ are listed in Table S5.

Table S3. Parameters of the shell-model potential for $SrTiO_3$ used in this work.

(a) Short-range potential					
Interaction	A (eV)	ρ (Å)	C_6 (eV Å^6)	r_{min} (Å)	r_{max} (Å)
O^{2-} shell - Sr^{2+} shell	1865.3676	0.348774	35.0	0	15
O^{2-} shell – Ti^{4+} shell	954.51433	0.372044	9.6	0	15
O^{2-} shell - O^{2-} shell	22764.3	0.149	43.0	0	15
(b) Shell model					
Species	Y (e)	k_2 (eV Å^{-2})	k_4 (eV Å^{-4})		
Sr^{2+} shell	1.526	7.8119652	50000		
Ti^{4+} shell	-1.000	879.96963	50000		
O^{2-} shell	-2.389	13.918477	50000		

Table S4. Performance of the shell-model potential in describing three phases of $SrTiO_3$ compared to experimental and DFT results.

$SrTiO_3$ phase		Energy (eV, per formula unit)	a	b	c	α	β	γ
Cubic	Potential	-150.1303	3.909	3.909	3.909	90	90	90
($Pm\bar{3}m$)	DFT[2]		3.910	3.910	3.910	90	90	90
	Expt.[3]		3.905	3.905	3.905	90	90	90
Tetragonal	Potential	-150.1351	5.524	5.524	7.818	90	90	90
(I4/mcm)	DFT[2]		5.503	5.503	7.829	90	90	90

	Expt.[4]			5.507	5.507	7.796	90	90	90
Rhombohedral	Potential	-150.1367		7.813	7.813	7.813	89.96	89.96	89.96
(R3c)	DFT[2]			7.802	7.802	7.802	89.92	89.92	89.92

Table S5. Parameters of the shell-model potential for $MgAl_2O_4$ used in this work.

(a) Short-range potential					
Interaction	A (eV)	ρ (Å)	C_6 (eV Å^6)	r_{min} (Å)	r_{max} (Å)
O^{2-} shell - Mg^{2+} shell	1273.2607	0.293297	0	0	15
O^{2-} shell –Al^{3+} shell	1474.400	0.3006	0	0	15
O^{2-} shell - O^{2-} shell	22764.3	0.149	20.37	0	15
(b) Shell model					
Species	Y (e)	k_2 (eV Å^{-2})	k_4 (eV Å^{-4})		
Mg^{2+} shell	1.585	361.7202	0		
Al^{3+} shell	1.458	588.6077	0		
O^{2-} shell	-3.00	59.487	0		

2.4 Spin density for SrO, MgO, and ZnO

In the main text, the spin density distributions of cation vacancies at different charge states are shown for $SrTiO_3$ and CeO_2, where O–O bonds or O–O–O chains are present. For $MgAl_2O_4$, only the isolated O–O bond configuration is stable at the neutral charge state. At charge state +1, both the O–O–O chain and the two-O–O configurations become stable. However, in this case, the system contains an even number of electrons, meaning that the excess charge cannot be observed in the spin density. Upon increasing the charge state further, the excess charge localizes on other atomic sites, as each O–O species can accommodate at most one excess hole. Consequently, unlike in $SrTiO_3$ and CeO_2, no spin density is observed for $MgAl_2O_4$ at any charge state.

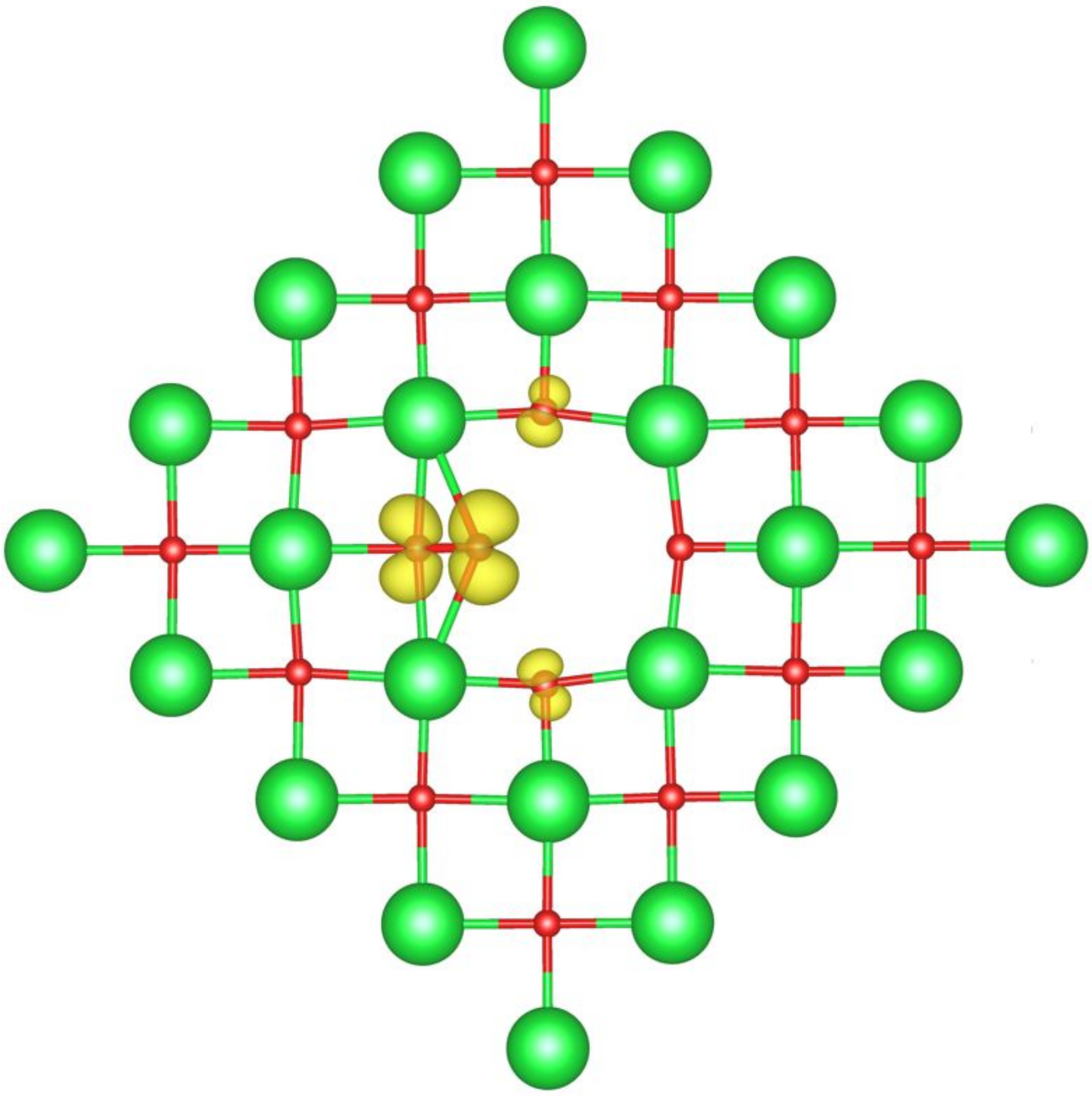

Figure S13. Spin density distributions of Sr vacancies with one O-O bond at charge states +1.

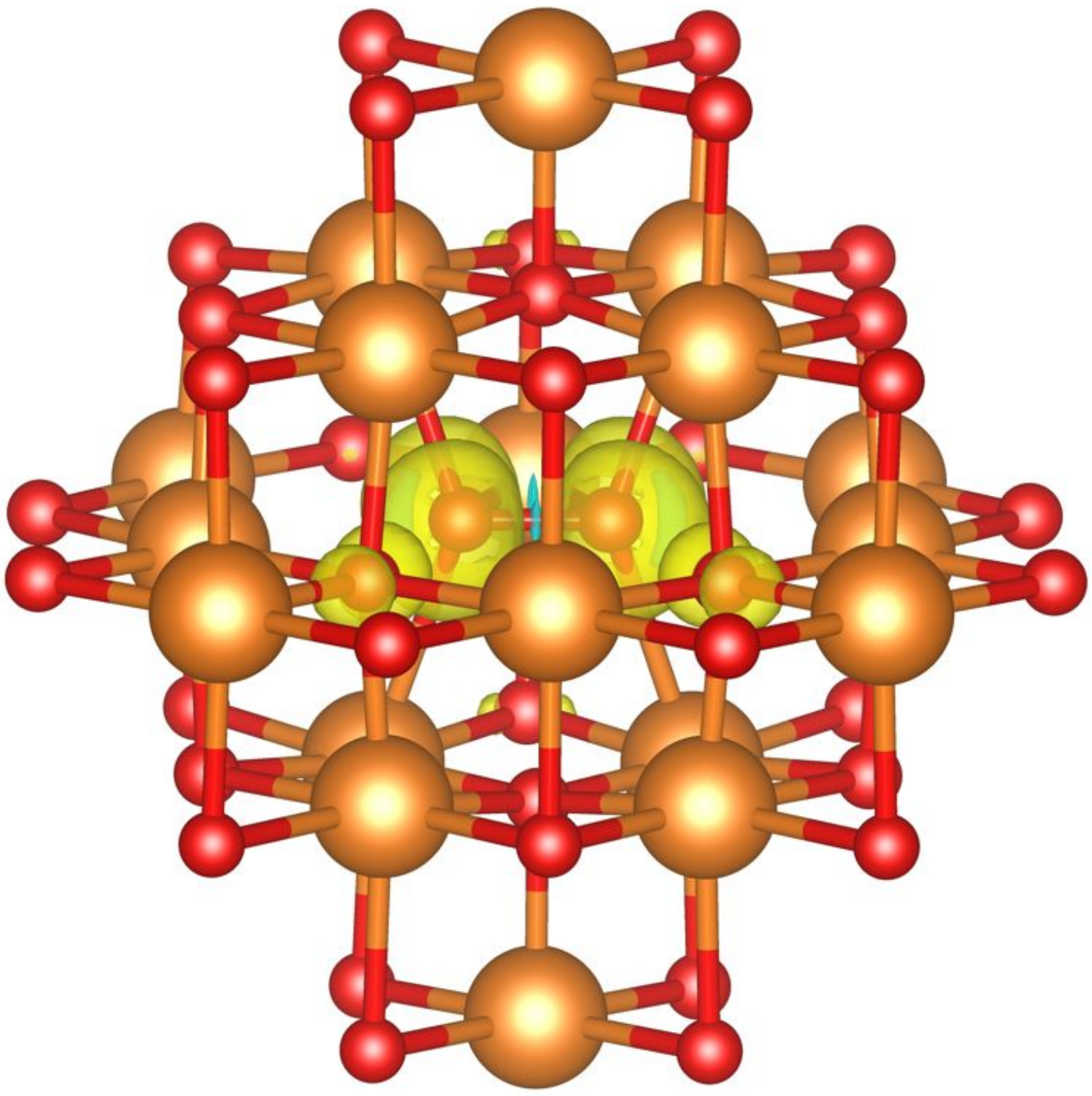

Figure S14. Spin density distributions of Mg vacancies with one O-O bond at charge states +1.

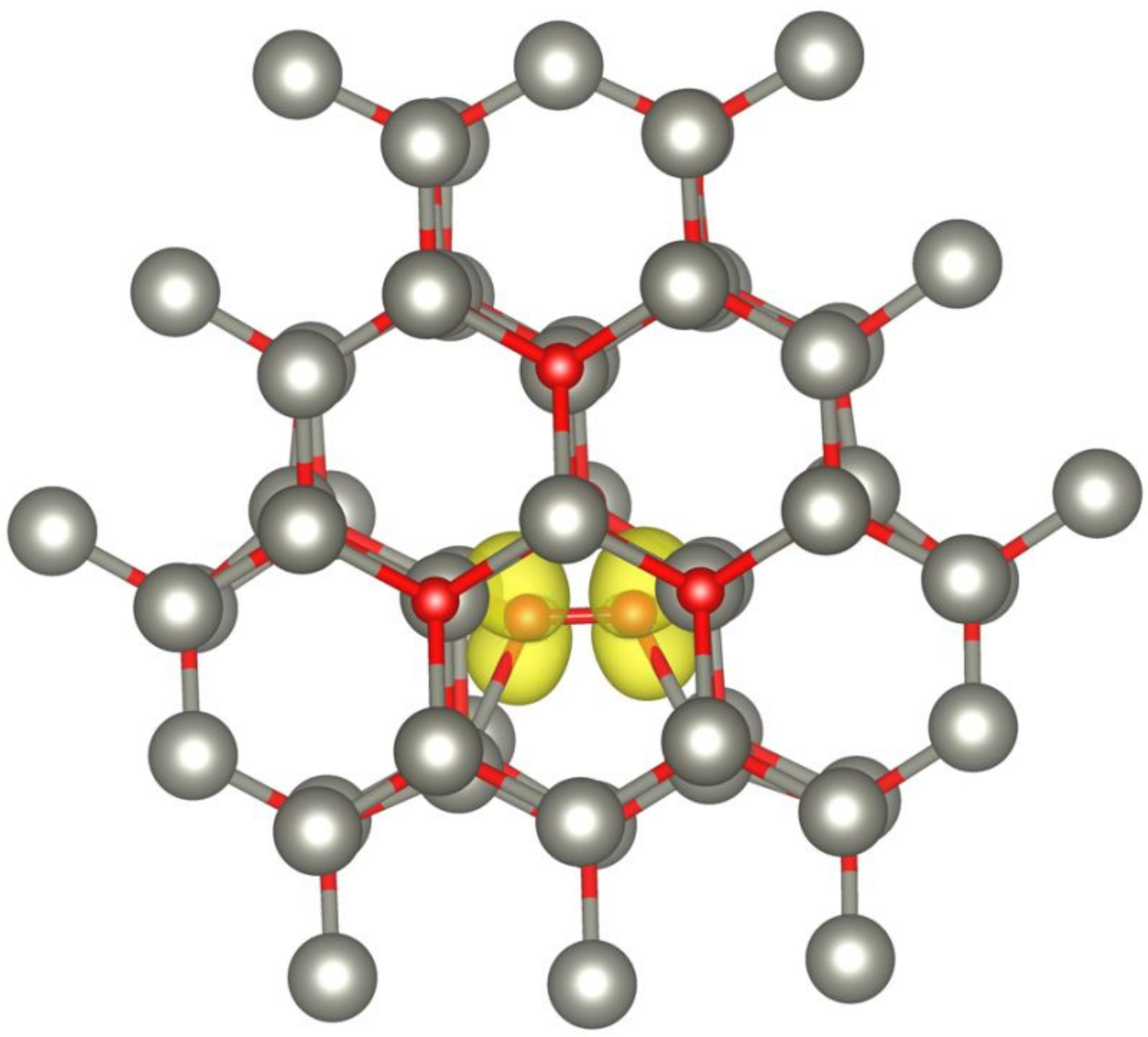

Figure S15. Spin density distributions of Zn vacancies with one O-O bond (case 1) at charge states +1.

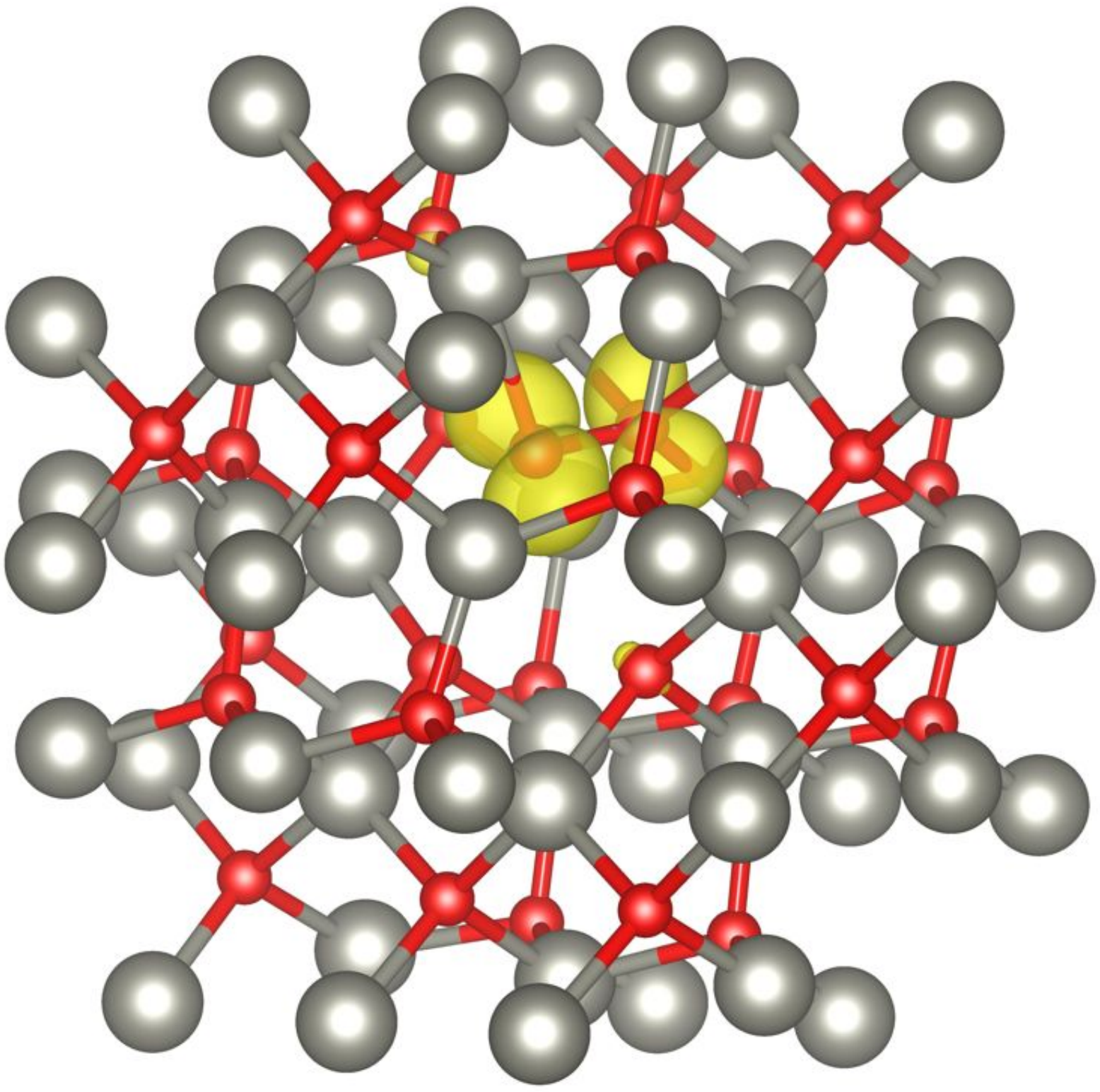

Figure S16. Spin density distributions of Zn vacancies with one O-O bond (case 2) at charge states +1.

3. Configurations contains O-O bonds or O-O-O chains by periodic density functional theory (DFT) calculations

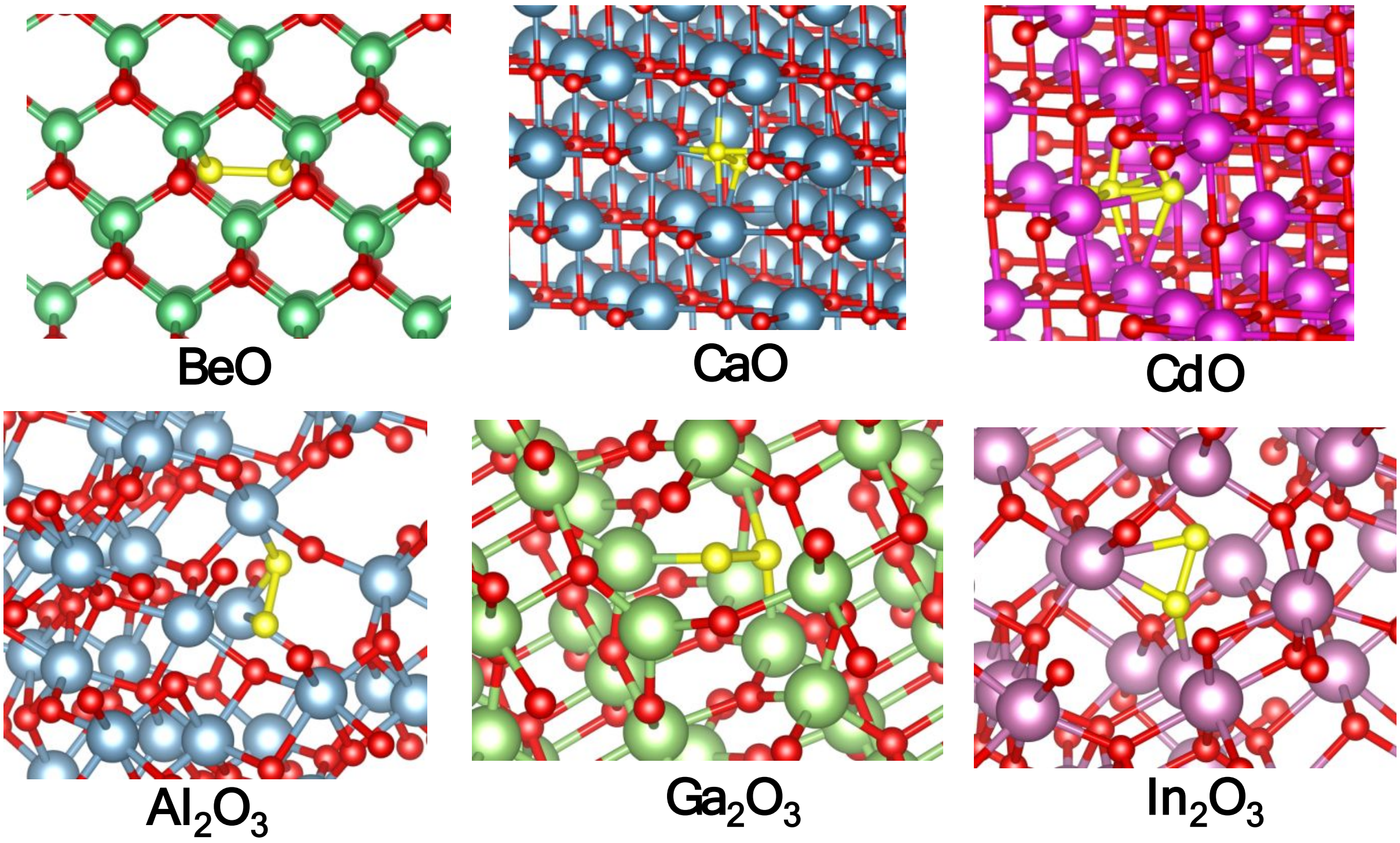

Figure S17. Reconstructed oxygen-bonded cation-vacancy configurations in BeO, CaO, CdO, Al_2O_3, Ga_2O_3, and In_2O_3. The small red spheres represent oxygen atoms, while the other spheres denote metal atoms. The oxygen atoms involved in the O–O bond are highlighted as yellow spheres.

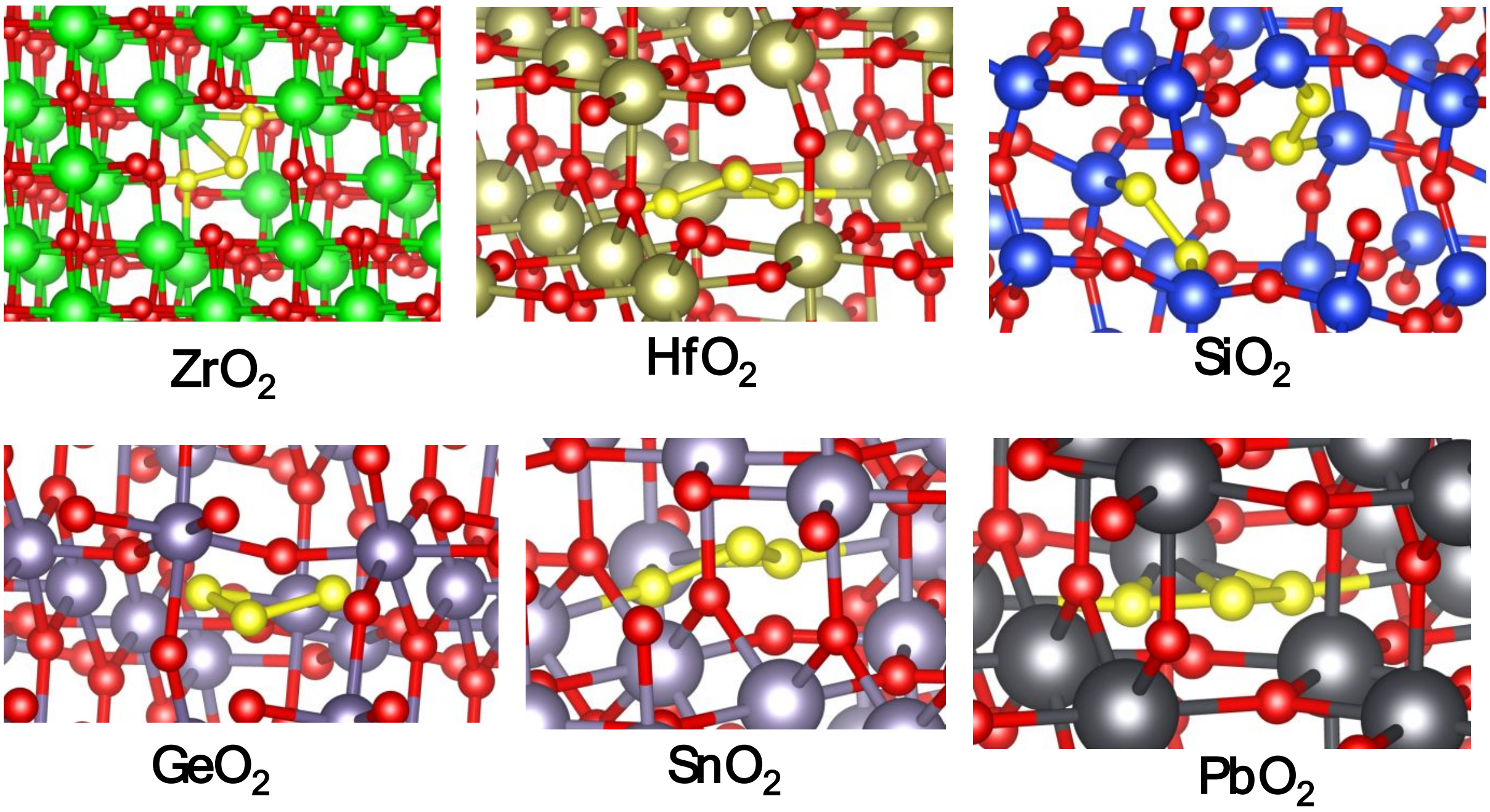

Figure S18. Reconstructed oxygen-bonded cation-vacancy configurations in ZrO_2, HfO_2, SiO_2, GeO_2, SnO_2, and PbO_2. The small red spheres represent oxygen atoms, while the other spheres denote metal atoms. The oxygen atoms involved in the O–O–O chains or O-O bonds are highlighted as yellow spheres.

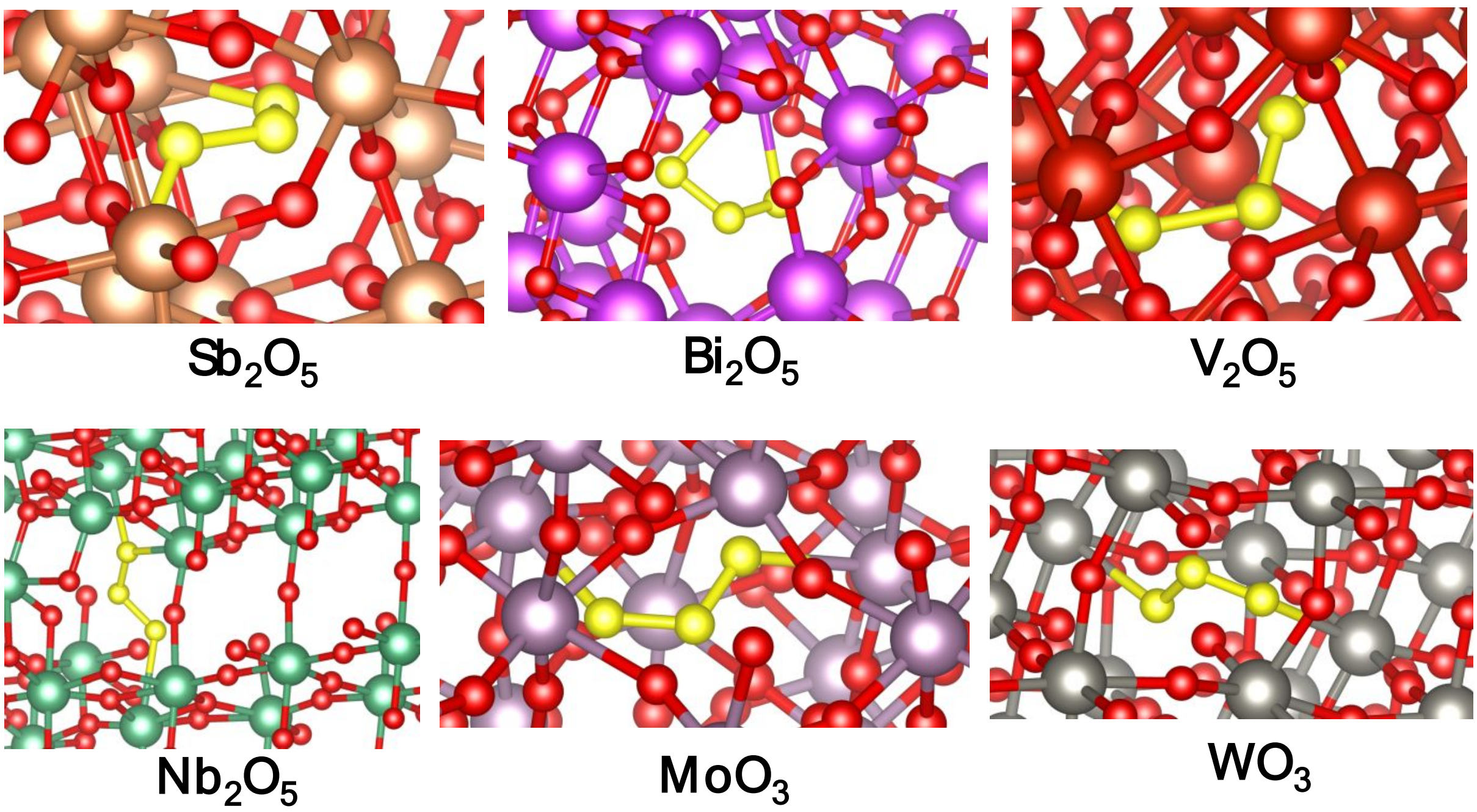

Figure S19. Reconstructed oxygen-bonded cation-vacancy configurations in Sb_2O_5, Bi_2O_5, V_2O_5, Nb_2O_5, MoO_3, and WO_3. The small red spheres represent oxygen atoms, while the other spheres denote metal atoms. The oxygen atoms involved in the O–O–O chains are highlighted as yellow spheres.

REFERENCES

1. Catlow, C. R. A.; Guo, Z. X.; Miskufova, M.; Shevlin, S. A.; Smith, A. G. H.; Sokol, A. A.; Walsh, A.; Wilson, D. J.; Woodley, S. M., Advances in computational studies of energy materials. *Philosophical Transactions of the Royal Society A: Mathematical, Physical and Engineering Sciences* **2010**, *368*, 3379-3456.
2. Smith, A. G. H. Structural and defect properties of strontium titanate. UCL (University College London), 2012.
3. Schmidbauer, M.; Kwasniewski, A.; Schwarzkopf, J., High-precision absolute lattice parameter determination of $SrTiO_3$, $DyScO_3$ and $NdGaO_3$ single crystals. *Structural Science* **2012**, *68*, 8-14.
4. Yuan, C.; Ye, S.; Xu, B.; Lei, W., Strain induced tetragonal $SrTiO_3$ nanoparticles at room temperature. *Appl. Phys. Lett.* **2012**, *101*.
